# Abscisic Acid Regulates Carbohydrate Metabolism, Redox Homeostasis and Hormonal Regulation to Enhance Cold Tolerance in Spring Barley

**DOI:** 10.3390/ijms241411348

**Published:** 2023-07-12

**Authors:** Junhong Guo, Gerrit T. S. Beemster, Fulai Liu, Zongming Wang, Xiangnan Li

**Affiliations:** 1 Key Laboratory of Black Soil Conservation and Utilization, Northeast Institute of Geography and Agroecology, Chinese Academy of Sciences, Changchun 130102, China; guojunhong@iga.ac.cn (J.G.); zongmingwang@iga.ac.an (Z.W.); 2 College of Advanced Agricultural Sciences, University of Chinese Academy of Sciences, Beijing 100049, China; 3 Laboratory for Integrated Molecular Plant Physiology Research (IMPRES), Department of Biology, University of Antwerp, Groenenborgerlaan 171, 2020 Antwerpen, Belgium; gerrit.beemster@uantwerpen.be; 4 Department of Plant and Environmental Sciences, Faculty of Science, University of Copenhagen, Højbakkegård Allé 13, DK-2630 Tåstrup, Denmark; fl@plen.ku.dk

**Keywords:** low temperature, abscisic acid, carbohydrate metabolism, 4D-proteomics, reactive oxygen species metabolism, *Hordeum vulgare*

## Abstract

Abscisic acid (ABA) plays a vital role in the induction of low temperature tolerance in plants. To understand the molecular basis of this phenomenon, we performed a proteomic analysis on an ABA-deficit mutant barley (*Az34*) and its wild type (cv Steptoe) under control conditions (25/18 °C) and after exposure to 0 °C for 24 h. Most of the differentially abundant proteins were involved in the processes of photosynthesis and metabolisms of starch, sucrose, carbon, and glutathione. The chloroplasts in *Az34* leaves were more severely damaged, and the decrease in Fv/Fm was larger in *Az34* plants compared with WT under low temperature. Under low temperature, *Az34* plants possessed significantly higher activities of ADP-glucose pyrophosphorylase, fructokinase, monodehydroascorbate reductase, and three invertases, but lower UDP-glucose pyrophosphorylase activity than WT. In addition, concentrations of proline and soluble protein were lower, while concentration of H_2_O_2_ was higher in *Az34* plants compared to WT under low temperature. Collectively, the results indicated that ABA deficiency induced modifications in starch and sucrose biosynthesis and sucrolytic pathway and overaccumulation of reactive oxygen species were the main reason for depressed low temperature tolerance in barley, which provide novel insights to the response of barley to low temperature under future climate change.

## 1. Introduction

Climate change triggers and exacerbates extreme weather events, including low temperatures, threatening crop productivity [1]. As a temperate plant species, spring barley (*Hordeum vulgare* L.) often suffers from low temperature stress, leading to a large reduction in grain yield. Low temperature causes the rigidification of membranes and damage to chloroplast structure, which directly depresses the photosynthetic electron transport, results in a burst of reactive oxygen species (ROS), and adversely affects plant growth [2,3,4]. Low temperature also disturbs the balance between the energy input and energy consumption, ultimately resulting in photoinhibition [5]. Moreover, low temperature typically suppresses the kinetics of metabolic reactions and results in inactivity of key enzymes [6,7,8].

Higher plants have developed several mechanisms to deal with low temperature stress at morphological, physiological, metabolic, and molecular levels [9]. Chloroplasts are the exclusive sites of photosynthesis and perform critical steps in a range of metabolic pathways, including the synthesis of pigments, lipids, and hormones [10]. Low temperature affects the functioning of chloroplasts by altering chloroplast ultrastructure and limiting chloroplast development, thereby inhibiting photosynthesis [4,11]. The photosystem complexes located in the thylakoid membrane can sense low temperatures [10], which reduce the efficiency of photosynthetic systems by rigidifying the thylakoid membrane and slowing down enzymatic reactions [10]. These changes result in decreased photosynthetic carbon assimilation in plants under low temperature.

Changes in protein relative abundance are closely related to the induction of low temperature tolerance, and many proteins have been reported to play a role in response to low temperature stress, mainly involving photosynthesis, ROS scavenging, carbohydrate metabolism, and hormonal signaling [12]. In *Arabidopsis thaliana*, 43 differentially abundant proteins under low temperature stress were identified that participate in photosynthesis, hormone biosynthesis, stress sensing, and signal transduction [13]. The expression of cold-responsive proteins and genes is regulated by different hormones, which interact with each other in a regulatory network [14,15,16]. Upon exposure to low temperature, plants enhance endogenous abscisic acid (ABA) levels by activating the synthetic pathway and/or inhibiting the pathway of degradation [17]. ABA and jasmonic acid (JA) interactively regulate the plant tolerance to abiotic stress, such as low temperature and drought, with JA acting upstream of ABA [3,18,19]. JA is an important upstream signal of the ICE-CBF/DREB1 pathway for promoting cold tolerance in *Arabidopsis* [20]. Salicylic acid (SA) plays a role in induction of cold tolerance by regulating ROS metabolism in wheat [21]. Low temperature induces SA biosynthesis through the isochorismate synthase pathway, leading to SA accumulation [22]. Nevertheless, how the hormonal regulatory network and the metabolisms of carbohydrate and ROS are functionally connected and induce low temperature tolerance remains largely elusive. Recently, 4-D proteomic has been widely applied in the research of plant stress responses. The 4-D proteomic is based on the high speed and sensitivity of the *timsTOF Pro* mass spectrometer (Brucker, Bremen, Germany), which combines PASEF (Parallel Accumulation Aerial Fragmentation) and TIMS (Trapped Ion Mobility Spectrometry) to repeatedly measure the collision cross section (CCS) of all detected ions, which can highly improve the quality and sensitivity of proteome [23,24,25].

Rapid temperature drops could lead to rapid modifications of protein synthesis. In cold shocked barley, cold response proteins are involved in modulating and protecting some cell functions when the temperature undergoes a sudden change [26]. In this study, wild type (WT) and ABA-deficient barley (*Az34*) were exposed to a 24-h low temperature at 0 °C to explore the role of ABA and downstream processes in the induction of low temperature responses. We performed 4D proteomics complemented with biochemical measurements of the activities of key enzymes involved in carbohydrate and ROS metabolisms using a semi-high throughput method, gene expression and hormonal profiling. We hypothesized that (i) the endogenous ABA would play key roles in the induction of low temperature response in barley, and (ii) the ABA induced modulation in carbohydrate and ROS metabolisms and hormonal regulations contribute to the induction of low temperature response in barley.

## 2. Results

### 2.1. Phenotypic Symptoms and Photosynthetic Electron Transport

**Figure 1:**
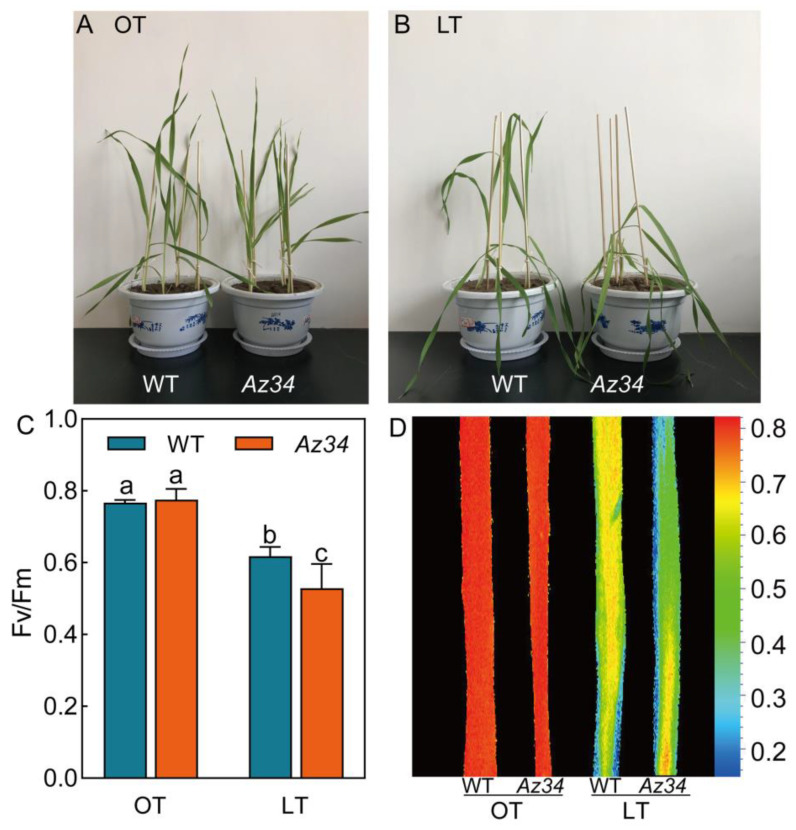
Phenotypic symptoms (**A**,**B**) and dark-adapted image of maximum quantum efficiency of photosystem II (Fv/Fm) (**C**,**D**) for the latest fully expanded leaf of wild type (WT) and *Az34* barley under optimum and low temperature. OT, optimum temperature; LT, low temperature. Different small letters indicate significant differences at *p* < 0.05 level. Data are expressed as means ± SEM (n = 3).

The growth rate of WT plants was slightly faster than that of *Az34* plants (Figure 1A). No significant difference in the photosynthetic electron transport was found between WT and *Az34* under the optimum temperature (Figure 1C,D; Table 1). A 24-h low temperature treatment at 0 °C adversely affected barley seedlings. More severe leaves wilted in *Az34* plants compared with WT plants after low temperature (Figure 1A,B). To determine the effect on photosynthesis we determined Fv/Fm as a measure of photosynthetic electron transport, which reflects the maximum quantum yield of PS II [27]. Fv/Fm of WT and *Az34* plants were significantly reduced 19.42% and 31.77% by exposure to low temperature, respectively. However, that of *Az34* was significantly lower (14.44%) than WT (Figure 1C,D). *An* and *g_s_* were both significantly reduced by low temperature in relation to the controls (Table 1). The *An* of WT and *Az34* plants were significantly reduced by 37.05% and 47.18% under low temperature. The *An* of *Az34* plants were significantly lower (16.40%) than WT plants under low temperature. The *g_s_* of WT and *Az34* plants were significantly decreased by 24.30% and 11.25% under low temperature. However, *g_s_* of *Az34* plants were 14.12% higher than WT plants under low temperature. Low temperature significantly reduced the maximum quantum yield for primary photochemistry (φP_O_) in both WT and *Az34* plants, while it was 14.52% lower in WT than in *Az34* plants (Table 1). Inversely, low temperature significantly decreased the probability that an electron moves further beyond than Q_A_ (ψE_O_) in *Az34* (21.54%), while did not affect that in WT (Table 1). Quantum yield for electron transport (φE_O_) was reduced to a similar extent in WT (27.27%) and *Az34* (37.25%) under low temperature. Interestingly, the quantum yield for reduction of end electron acceptors at the PSI acceptor side (φRo) was increased by 52.63% in WT, whilst it was decreased by 46.15% in *Az34* under low temperature (Table 1). This suggested that the photosynthesis of *Az34* is more susceptible to low temperature than WT.

### 2.2. Proteomics

**Figure 2:**
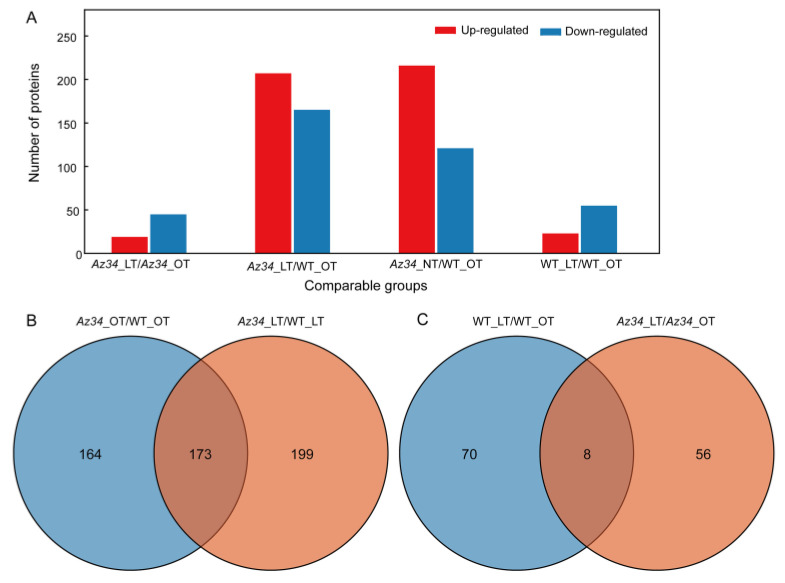
Differentially expressed proteins identified by 4D proteomics in the latest fully expanded leaf of wild type (WT) and *Az34* barley under optimum and low temperature. (**A**) Number of up- and down-regulated proteins in *Az34* compared with WT under optimum and low temperature, respectively. (**B**) Venn diagram showing the number of differentially expressed proteins in *Az34* compared with WT under different temperature treatments. (**C**) Venn diagram showing the number of differentially expressed proteins in low temperature compared with optimum temperature in WT and *Az34*, respectively. OT, optimum temperature; LT, low temperature.

To obtain a genome-wide view of the effects of low temperature stress and the role of ABA signaling on protein expression profiles, we performed a 4D-proteomic analysis on the latest fully expanded leaf in *Az34* and WT plants under both temperature regimes. The quality of the protein extracts was demonstrated by SDS-PAGE (Figure S2). A total of 3879 proteins were identified based on the barley protein database (Uniprot *Hordeum vulgare*); of those, 3200 proteins were quantified (Table S1). Proteins that showed statistically significant (*p* < 0.05) differences that exceeded 1.5-fold in abundance between two genotype/treatment combinations were identified as differentially abundant proteins (DAPs). Under optimum temperature, 337 DAPs were identified in *Az34* compared with WT, including 216 up-regulated and 121 down-regulated proteins. Comparing the differentially expressed proteins in *Az34* and WT plants in response to low temperature shows that roughly four times as many proteins are differentially expressed between the genotypes under both conditions then in response to low temperature. Under LT 372 DAPs were identified between the genotypes, including 207 up-regulated and 165 down-regulated in *Az34* (Figure 2A). There was about 1/3 overlap among DAPs in WT compared with *Az34* under the two different temperature conditions (Figure 2B). DAPs in low temperature compared with optimum temperature in WT and *Az34*, respectively, show only limited overlap (Figure 2C). Overall, these results indicate a strong difference between the two genotypes and their response to low temperature. Subcellular localization analysis showed that the DAPs were overrepresented for proteins located in the chloroplasts (Figure S3). 

We then performed overrepresentation analysis for the 337 DAPs in *Az34* compared to WT plants under optimum temperature. Overrepresented biological processes included cellular metabolic, stress response, and endogenous stimulus responding pathways (Figure S4). Among them, lipoxygenase, glucose-6-phosphate 1-dehydrogenase, cell wall invertase, vacuolar invertase, and glutamate synthase were up-regulated, while photosynthetic NDH subunit of subcomplex B1, photosynthetic NDH subunit of subcomplex B4, NAD(P)H-quinone oxidoreductase subunit 1, FMN_dh domain-containg protein, peroxidase, glutathione peroxidase, aldehyde oxidase 3, and sucrose synthase were down-regulated in *Az34* compared with WT under optimum temperature. Functional annotation of the DAPs mainly enriched Golgi-associated and COPI-coated vesicle proteins, membrane coat proteins, and membrane protein complex in *Az34* plant compared with WT plant under optimum temperature (Figures S4 and S5). In addition, the molecular functions enrichments mainly included galactosyltransferase activity, GTPase regulator activity, GTPase activator activity, and glutamine-tRNA ligase activity.

Similarly, we analyzed the DAPs between *Az34* with WT under low temperature. These were enriched for the biological process terms cellular metabolic process (105 proteins), organic substance metabolic process (89 proteins), primary metabolic process (69 proteins), nitrogen compound metabolic process (64 proteins), chemical response (55 proteins), biosynthetic process (53 proteins), stress response (53 proteins), and abiotic stimulus response (46 proteins, Figure S4C). The cellular components for DAPs mainly included intracellular (198 proteins), intracellular organelle (167 proteins), membrane-bounded organelle (160 proteins), photosynthetic membrane (37 proteins), and plasma membrane (35 proteins, Figure S4C). In addition, the enrichment analysis showed that the main categories, “photosystem II repair”, “cell wall pectin metabolic process”, “mRNA catabolic process”, “chloroplast thylakoid membrane protein complex”, “UDP-glucose 6-dehydrogenase activity”, “cytochrome-c peroxidase activity”, and “peroxidase activity” were all enriched in *Az34* compared with WT under low temperature (Figure S5). KEGG pathway enrichment analysis indicated that the pathways of photosynthesis, glutathione metabolism, and ascorbate and aldarate metabolism were significantly down-regulated, while the pathways of valine, leucine, and isoleucine biosynthesis, pantothenate, and CoA biosynthesis and histidine metabolisms were significantly up-regulated in *Az34* in relation to WT under low temperature (Figure S5).

**Figure 3:**
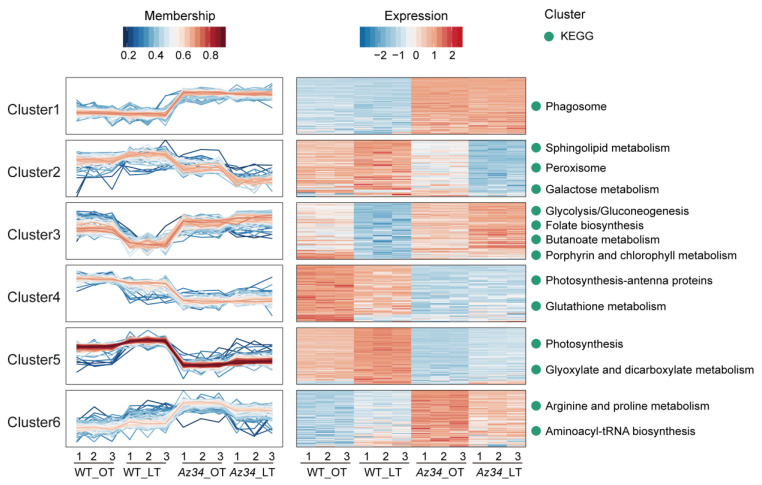
Clustering analysis of differentially abundant proteins (DAPs) between the four comparable groups (WT_LT/WT_OT, *Az34*_LT/WT_OT, *Az34*_LT/*Az34*_OT, *Az34*_NT/WT_OT). Mfuzz was used to cluster a total number of 472 DAPs with significantly levels and heatmap of 472 DAPs and key KEGG pathways. OT, optimum temperature; LT, low temperature. Data are expressed as means ± SEM (n = 3).

We further analyzed all DAPs in the four comparable groups, that is *Az34* compared with WT under two temperature regimes, respectively. The proteins not identified in all biological replicates had to be excluded. In total, 472 proteins were significantly affected in four comparable groups. These proteins clustered into six patterns (Figure 3; Table S2) using the packages Mfuzz and complete cluster by RStudio. To further analyze the metabolism pathways in each cluster, we separately conducted the KEGG pathway enrichment analysis of the proteins in each cluster. Cluster 1 contained 90 proteins with higher expression levels in *Az34* compared with WT under two temperature regimes, where the category “phagosome” was significantly enriched. Cluster 2 contained 76 proteins, which were up-regulated under low temperature as compared to that under optimum temperature in WT plants, whereas they were down-regulated in *Az34* plants as compared to WT plants under low temperature. The pathways of “sphingolipid metabolism”, “peroxisome”, and “galactose metabolism” were enriched in Cluster 2. Cluster 3 contained 84 proteins, which were down-regulated under low temperature as compared to optimum temperature in WT plants, whereas they were up-regulated in *Az34* plants compared to WT plants under low temperature. The pathways of “glycolysis/gluconeogenesis”, “folate biosynthesis”, “butanoate metabolism”, and “porphyrin and chlorophyll metabolism” were enriched in Cluster 3. Cluster 4 and Cluster 5 showed opposite patterns in response to low temperature in WT plants. However, the protein abundance was significantly lower in *Az34* plants than WT plants both in Cluster 4 and Cluster 5. The pathways of “photosynthesis-antenna proteins” and “glutathione metabolism” were enriched in Cluster 4, while the pathways of “photosynthesis” and “glyoxylate and dicarboxylate metabolism” were enriched in cluster 5. Cluster 6 contained 86 proteins with higher abundance under low temperature in relation to the optimum temperature in WT plants. In addition, *Az34* plants possessed higher protein abundance than WT plants under low temperature, where the pathways of “arginine and proline metabolism” and “aminoacyl-tRNA biosynthesis” were enriched (Figure 3).

**Figure 4:**
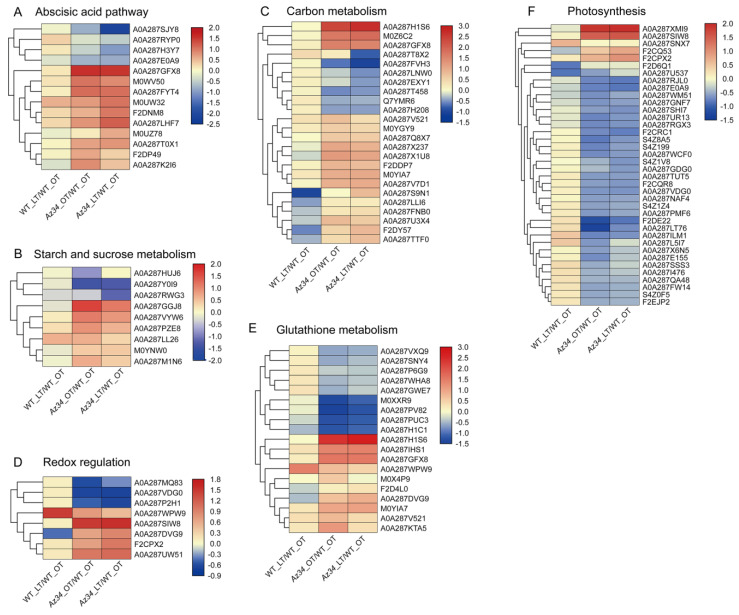
Heat map of differential expressed proteins related to the metabolisms of abscisic acid (**A**), starch and sucrose (**B**), carbohydrate (**C**), reactive oxygen species (**D**), glutathione (**E**), and photosynthesis (**F**) in the latest fully expanded leaf in wild type and *Az34* compared with WT under optimum temperature, respectively. The analysis was performed by TBtools and the data was presented by log two-fold change. Red and blue indicates increase and decrease in protein expression, respectively. OT, optimum temperature; LT, low temperature. Data are expressed as means ± SEM (n = 3).

In addition, we performed cluster analysis with the method of Euclidean distance and complete cluster by Tbtools software (v0.67361) [28] for the major pathways (Figure 4, Table S3). Most of the proteins related to the photosynthesis and glutathione metabolism were down-regulated in *Az34* compared to WT. In *Az34*, aldehyde oxidase 3 (A0A287SJY8) involved in ABA biosynthesis was down-regulated compared with WT, which decreased the ABA concentration in *Az34*. However, aldehyde oxidase 3 was up-regulated under low temperature compared to optimum temperature in *Az34*. It is implied that low temperature induces the accumulation of ABA. The pathways of redox regulation and metabolism of starch and sucrose were differently affected by low temperature in *Az34* and WT. Therefore, these results have suggested that ABA deficiency altered the response to low temperature stress by changing the expression of proteins related to sphingolipid metabolism, peroxisome, glycolysis, porphyrin and chlorophyll metabolism, photosynthesis-antenna proteins, glutathione metabolism, photosynthesis, and redox regulation.

### 2.3. Chloroplast Ultrastructure

**Figure 5:**
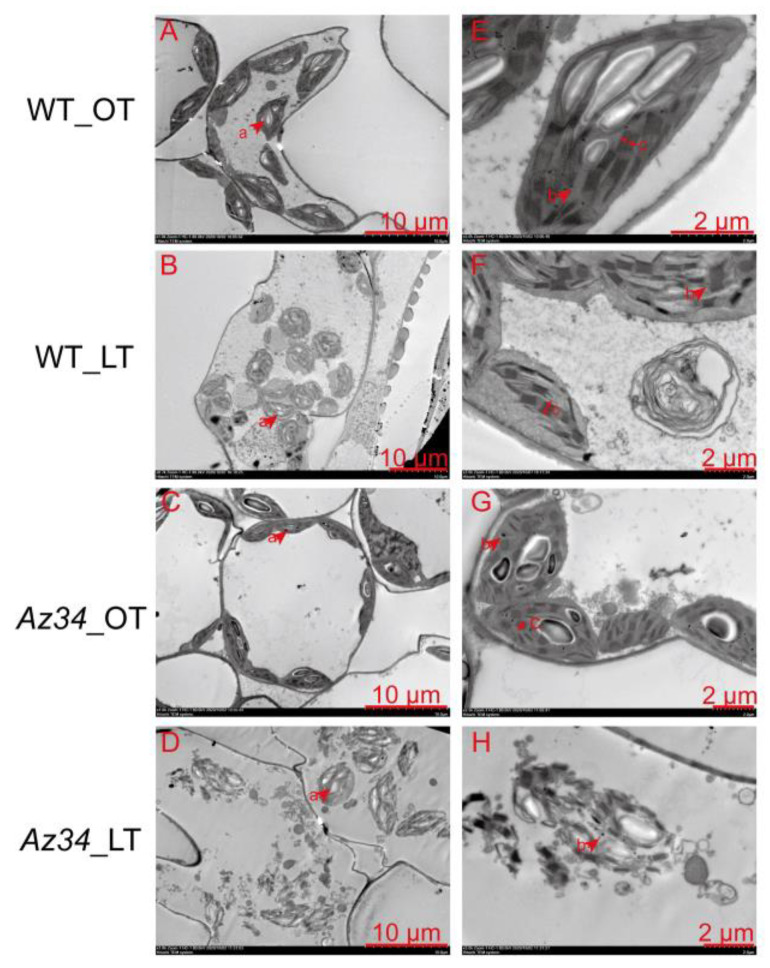
Ultrastructure of chloroplasts in the latest fully expanded leaf of wild type (WT) and *Az34* barley under optimum and low temperature. (**A**,**E**) Ultrastructure of chloroplasts in WT under optimum temperature; (**B**,**F**) Ultrastructure of chloroplasts in WT under low temperature; (**C**,**G**) Ultrastructure of chloroplasts in *Az34* under optimum temperature; (**D**,**H**) Ultrastructure of chloroplasts in *Az34* under low temperature; OT, optimum temperature; LT, low temperature; a, starch grain; b, grana lamellae; c, osmiophilic lipid droplets. (**A**–**D**): bar = 10 μm; (**E**–**H**): bar = 2 μm.

Because the expression of proteins related to thylakoid was remarkably regulated in *Az34* under low temperature (Figure S5), we set out to analyze the effect of the low temperature on chloroplast ultrastructure in the latest fully expanded leaf of WT and *Az34*. The TEM images showed that under optimum temperature the chloroplasts had well-developed thylakoid systems, complete external envelope, and clear boundary in WT and *Az34* plants (Figure 5). The chloroplast ultrastructure was adversely affected by low temperature in both WT and *Az34*. The chloroplasts in *Az34* were more severely damaged by low temperature compared to those of WT, and we consistently found some chloroplasts that were broken in *Az34* leaves, but not in those of WT. In addition, at higher magnification (bar = 2 μm), the images showed that the grana lamellae thickness was greater in WT than that in *Az34* under low temperature.

### 2.4. Carbohydrate and ROS Metabolisms

**Figure 6:**
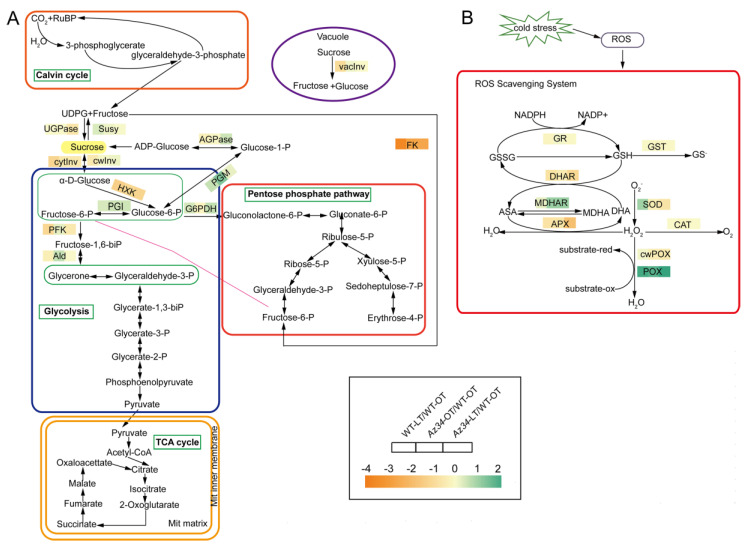
Heatmap of activities of key carbohydrate metabolism (**A**) and antioxidant (**B**) enzymes in the latest fully expanded leaf of wild type (WT) and *Az34* barley under optimum and low temperature. Ald, aldolase; UGPase, UDP-glucose pyrophosphorylase; Susy, sucrose synthase; AGPase, ADP-glucose pyrophosphorylase; HXK, hexokinase; PGI, phosphoglucomutase; G6PDH, glucose-6-phosphate dehydrogenase; PFK, phosphofructokinase; PGM, phosphoglucomutase; FK, fructokinase; vacInv, vacuolar invertase; cytInv, cytoplasmic invertase; cwInv, cell wall invertase; SOD, superoxide dismutase; CAT, catalase; APX, ascorbate peroxidase; POX, cytoplasmic peroxidase; cwPOX, apoplastic peroxidase; GST, glutathione S-transferase; MDHAR, monodehydroascorbate reductase; GR, glutathione reductase; DHAR, dehydroascorbate reductase. OT, optimum temperature; LT, low temperature. The difference of activity for a given enzyme among these treatments was deviation standardization and converted to a color scale. Data are expressed as means ± SEM (n = 3) and was transformed by log 2-fold change.

The proteomic data indicated that the major pathways modulated by ABA-deficiency and low temperature were related to the carbohydrate and ROS metabolisms. Therefore, we further determined the activities of key enzymes in carbohydrate and ROS metabolisms. Low temperature stress significantly increased the activities of two carbohydrate metabolism enzymes (i.e., G6PDH and PGM) while it reduced the activities of UGPase, HXK, Ald, PFK, vacInv and cytInv, in relation to the optimum temperature control (Figure 6A; Table 2; Figure S6). Low temperature stress significantly reduced the activities of three invertases (cytInv, cwInv and vacInv) in WT; however, the activities of these three invertase were not affected in *Az34* (Figure 6A and Figure S6). The activity of invertases plays an important role in the accumulation of sugars under low temperature [4]. Therefore, we tested the concentration of soluble sugars, and the results showed that low temperature stress significantly affected the accumulation of total soluble sugars in WT and *Az34* by changing the activities of invertase, HXK and UGPase (Figure S8A). In addition, low temperature significantly increased PGM activity in WT, while it was opposite in *Az34*. These results implied that the reduced ability of osmotic adjustment in *Az34* is related to sucrolytic and glycolysis under low temperature.

In the antioxidant metabolism low temperature significantly increased SOD activity in WT, while it was not affected in *Az34* (Figure 6B; Table 3; Figure S7). The POX activity was significantly decreased by low temperature in WT, while in *Az34* the induction of POX activity was absent. In addition, low temperature stress markedly decreased the activities of APX and DHAR in WT plants. In *Az34*, however, the induction of APX and DHAR activities were absent. Low temperature stress had no effect on the activities of CAT, GST, GR, and cwPOX in WT or *Az34* plants. To investigate the effects of altered antioxidant activities, we determined the concentration of H_2_O_2_, and the results showed that under low temperature, *Az34* has higher H_2_O_2_ levels than WT (Figure S8E). These results suggested that the reduced ability of the *Az34* mutant to scavenge ROS correlates with impaired induction of SOD and POX enzyme activity and H_2_O_2_ accumulation under low temperature stress.

### 2.5. Hormonal Regulatory Network

**Figure 7:**
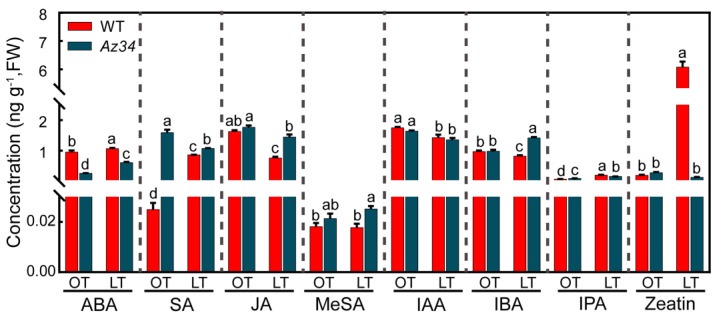
Concentrations of key hormones in the latest fully expanded leaf of wild type (WT) and *Az34* barley under optimum and low temperature. ABA, abscisic acid; SA, salicylic acid; MeSA, methyl salicylic acid; JA, jasmonate; IAA, indole-3-acetic acid; IBA, indole-3-butytric acid; IPA, indolepro pionic acid. OT, optimum temperature; LT, low temperature. Different small letters indicate significant differences at *p* < 0.05 level. Data are expressed as means ± SEM (n = 3).

To investigate the interaction of ABA and other hormones in barley under low temperature, we determined the concentrations of hormones in leaves in WT and *Az34*. The concentrations of seven key hormones were significantly affected by low temperature, except for MeSA, which belongs to SA (Figure 7; Table 4). As expected, the *Az34* plants possessed significantly lower ABA concentration than WT under optimum and low temperatures. Nevertheless, the ABA concentration was significantly increased by 11.58% and 134.62% under low temperature in both WT and *Az34*, respectively. It was noted that the ABA concentration significantly was 42.45% lower in *Az34* plants than WT plants under low temperature. The SA concentration was significantly increased by low temperature in WT but decreased in *Az34*. Under low temperature, the SA concentration was 24.42% higher in *Az34* plants than WT plants. Levels of IPA, a kind of auxin, was significantly increased by low temperature in WT and *Az34* (197.06% and 73.91%), respectively. The level of IPA was 20.79% lower in *Az34* than WT under low temperature. The JA concentration was significantly decreased in WT and *Az34* (53.09% and 17.61%) after low temperature treatments, respectively. After low temperature treatment, JA concentration was 90.79% higher in *Az34* than WT. Under low temperature, the IBA concentration was significantly reduced (15.46%) in WT but increased (43.88%) in *Az34*. In addition, the concentration of zeatin, a member of cytokines, was strongly increased by 29.4 times by low temperature in WT but reduced by 53.57% in *Az34*. These results show that altered ABA levels in the mutant affect the levels and response of other hormonal pathways under low temperature, which may be correlated with low temperature tolerance in barley.

### 2.6. Principal Component Analysis

Here, the PCA used two principal components (PC) and liner combination of variables to take into a high amount of variance in the dataset (Figure S9). The structure of observation among different terms of treatment was changed under low temperature (Figure S9). The ABA and SOD activity were closely related to the response of WT plants to low temperature, while the activities of FK, MDHAR, and AGPase were closely related to the response of *Az34* plants to low temperature. The outputs of PCA indicated that ABA was negatively correlated to *g_s_* while positively correlated to the activities of SOD and UGPase. In addition, ABA was correlated negatively to photosynthetic parameters (φPo, *An*, Fv/Fm and φEo) while negatively to carbohydrate metabolism enzyme activities (AGPase, FK, MDHAR, cwInv, and vacInv).

## 3. Discussion

Plants respond to low temperature stress at various levels, including physiological and molecular processes [29]. To survive low temperature stress, plants have developed many protective mechanisms, including ultrastructural reorganization of chloroplasts, modifications of carbohydrate metabolism, and activations of antioxidant systems [4,27,30,31]. 

### 3.1. ABA Deficiency Increased the Sensibility of Photosynthetic System and Chloroplast Ultrastructure to Low Temperature

The common phenotypic symptoms are leaf wilting and even death under low temperature [4]. Here, the barley plants exposed to low temperature showed a clear symptom of leaf wilting, particularly the *Az34* plant, which was severely damaged (Figure 1). Leaf is the main organ for photosynthesis, and the negative effects of low temperature on leaf directly limit crop growth and productivity [8]. Here, it was found that low temperature inhibited the *An* and *g_s_* both in WT plants and *Az34* plants. *An* was reduced due to stomatal closure induced decrease in photosynthetic CO_2_ uptake [32], which may be driven by changes in ABA level. Previous studies have shown that the concentration of ABA was significantly increased by low temperature [33], which may contribute to low temperature response. Low temperature reductions adversely affects barley growth by negatively affecting the photosynthesis [34,35]. 

Chlorophyll fluorescence has been widely used to investigate the photosynthetic electron transport as influenced by low temperature [36,37,38]. The efficiency of the light reactions, which is expressed as φPo [27], was lower both in WT and *Az34* plants under low temperature in relation to that under optimum temperature. When exposed to low temperature, the efficiency balance of the dark reactions after Q_A_, which is reflected by ψEo, was lower. The φEo reveals the maximum quantum yield for electron transport beyond Q_A_ and also indicates the maximum quantum yield of primary photochemistry, while φRo represents the quantum yield for reduction of end electron acceptors at the PS I acceptor side (RE) [27]. Previous studies have reported the close correlation between φRo and net photosynthesis rate under various light conditions [39,40,41]. Here, ABA-deficiency resulted in a large reduction in φRo under low temperature, indicating that ABA deficiency has caused more severe photoinhibition of PS I under low temperature and the possibility of secondary damages, which was in line with the previous studies [41,42,43]. In addition, the two parameters were decreased under low temperature in relation to that under optimum temperature in WT and *Az34*, suggesting that low temperature caused damage to the primary photochemistry reaction and electron transport, thereby reducing the photosynthetic efficiency, as exemplified by the lowered *A_n_*. The structure of photosynthetic apparatus is also crucial for sustaining photosynthetic rates [44]. Under low temperature, the grana and thylakoid membranes decompose, resulting in a decrease in chlorophyll concentration [45]. In agreement with this, here, the grana lamellae were reorganized in chloroplasts, and the number of grana lamellae was lower under low temperature, compared with that under optimum temperature. In addition, the ultrastructure of chloroplast was more severely damaged in *Az34* than WT under low temperature. This could be linked to the lower ABA concentration in *Az34*, which failed to sustain the fluidity of chloroplast membrane and maintain the ultrastructure of chloroplasts. Similarly, it has been shown that the ABA deficiency can reduce the induction of low temperature tolerance in barley by degrading the chloroplasts [45]. According to our proteome analysis, the processes involved in light reaction, sugar metabolism, and redox regulation were closely related to the different responses of WT and *Az34* plants to low temperature. Bioinformatic analysis showed that 178 differently expressed proteins were localized in chloroplasts (Figure S3), suggesting that photosynthesis was a key process affected by low temperature damage. Here, KEGG analysis showed that the photosynthesis and metabolisms of ascorbate, aldarate, and glutathione were down-regulated in *Az34* in relation to WT under low temperature, implying that ABA deficiency is associated with a reduced ability of low temperature response in barley plants.

### 3.2. ABA Induced Modifications in Metabolisms of Carbohydrate and ROS Contributed to Low Temperature Tolerance

Photosynthesis produces carbohydrates, which are major energy resources that are associated with low temperature response in higher plants [46]. Many carbohydrate metabolism enzymes are involved in this process. Here, the activity of key carbohydrate metabolism enzymes was significantly affected by low temperature and ABA deficiency in the latest fully expanded leaf as shown in the heat map. This is accordance with other studies, which reported that ABA can change the activities of carbohydrate enzymes and carbohydrate levels under stress conditions [47,48,49]. The activities of the three invertases were significantly decreased in WT but not affected in *Az34* under low temperature. It has been reported that vacInv and cwInv are key metabolic enzymes involved in the response of plants to adverse environment [50], and the changes of activities of these enzymes could be modulated by ABA levels [51]. This was also the case in the present study. It has been well documented that the most fundamental function of glycolysis is to provide energy and precursors for anabolic process, which connects the carbohydrate metabolism and growth [52]. Pyruvate (PK), HXK, and PFK are key enzymes in glycolysis, which play important roles in carbohydrate utilization to provide energy for plant growth and development [53]. Among these enzymes involved in glycolysis, HXK catalyze hexosaccharide phosphorylation acts as a hexose sensor in sugar signal transduction and response to abiotic stresses [54]. Here, the activity of HXK decreased in WT plants, while it was not changed in *Az34* plants under low temperature. HXK, a sugar-phosphorylating enzyme, plays a role in guard cells, stimulating stomatal closure in response to sugar levels [55]. Combined with our results, this may indicate that ABA deficiency has an adverse effect on glycolysis, and might even be inhibited to regenerate ATP for cellular processes under low temperature conditions [56]. In most plants, sucrose is the major transport sugar, and it is channeled into different subcellular compartments through various pathways [57]. As a source of carbon and energy, sucrose is broken down into hexoses catalyzed by sucrose synthase or invertase [58]. The UGPase enzyme plays a vital role in the process of sucrose synthesis and biosynthesis of the cell wall [59]. In the present study, *Az34* mutant has a lower UGPase activity than WT, which may affect the sugar metabolism and osmotic adjustment under low temperature. Overall, our proteome analysis suggested that endogenous ABA levels affect the sugar metabolism by changing carbohydrate assimilation related proteins under low temperature. 

Low temperature stress induced overproduction of ROS results in oxidative damage and cell membrane injury [17]. Antioxidant enzymes play vital roles in ROS scavenging; among them, SOD catalyzes the disproportionation of singlet oxygen (^1^O_2_) and produces H_2_O_2_, and H_2_O_2_ is decomposed to H_2_O and O_2_ by CAT and APX [60]. In our study, *Az34* plants possessed lower SOD activity than WT plants under low temperature, which indicated that ABA deficiency leads to accumulate superoxide anion. Under low temperature, there is an increase in the H_2_O_2_ accumulation in chloroplasts, leading to cellular damage and destruction [61]. Here, low temperature induced the H_2_O_2_ accumulation both in WT and *Az34* plants, and it was higher in *Az34* than WT. This illuminated that ABA deficiency has a detrimental effect on ROS scavenging system under low temperature stress, which is consistent the previous studies from Yu et al. [62] and Huang et al. [17], where ABA reduces oxidative damage by increasing the activity of antioxidant enzymes under low temperature stress. APX catalases the conversion of H_2_O_2_ to H_2_O and O_2_, playing a crucial role in decreasing the phytotoxic effects of H_2_O_2_ in plants [63]. In our study, APX activity in *Az34* plants was lower than WT plants by low temperature, which is consistent with the study of Hong et al. [64], who reported that ABA deficiency suppresses *OsAPx8* expression and APX activity under NaCl stress [64]. DHAR catalyzes dehydroascorbate (DHA) to ascorbate (AsA) in AsA-GSH cycle, which plays a key role in low temperature response [65]. Here, the activity of DHAR was significantly decreased in WT plants under low temperature, probably leading to a lower AsA level [65]. In line with this, an earlier study documented that higher levels of ABA enhance cold tolerance by increasing the activities of CAT, SOD, POD, APX, DHAR, and MDHAR in wheat leaves [62]. These results suggested that endogenous ABA level might be involved in regulating the AsA-GSH cycle, thereby affecting the low temperature response.

### 3.3. ABA Deficiency Induced Changes in Hormonal Regulatory Network Were Related to Low Temperature

ABA plays important roles in plant response to low temperature stress [66]. Here, the phenotype and physiological responses indicated that *Az34* plants had reduced ABA levels and were more susceptible to low temperature than WT plants. It was suggested that ABA-dependent signaling pathways are involved in the induction of low temperature tolerance. This could be due to the fact that ABA deficiency decreases the transport of sucrose from source to sink, hence inhibiting the root growth [67]. It could be at least partly due to the changes in long-distance transports of sugar, though the sugar concentrations in roots and shoots and the gene expressions of transporters in *Az34* plants were not affected by low temperatue. In addition, it illuminated that ABA-dependent signaling pathways are involved in the induction of low temperature tolerance. JAs also affect low temperature tolerance by regulating C-repeat binding factor (CBF) pathway [67]. Our study showed that the JA concentration was decreased both in WT and *Az34* under low temperature, suggesting that ABA downregulates JA. A previous study reported that JA functions in the downstream of ABA to activate CBF pathway in light quality mediated cold tolerance [68,69]. Here, ABA deficiency enhanced the JA level under low temperature conditions, suggesting ABA downregulates JA accumulation. It has been suggested that JAs also can activate the plant defense mechanisms in response to low temperature [67]. Based on our study, it is likely that ABA deficiency might play a negative role in the process of JA regulates CBF pathway under to low temperature. There is an antagonistic crosstalk between ABA-mediated signaling and SA-mediated signaling in environmental stress responses [17,46,70]. ABA deficiency caused the accumulation of SA in the present study, which was similar with the previous results that ABA/PYR1 signaling could activate the SA biosynthesis [71]. The crosstalk between ABA signaling and SA signaling under low temperature should be further studied. Zeatin, a kind of cytokinin, plays a key role in plant growth and development. Our study showed that the zeatin concentration was significantly increased by low temperature in WT, along with the previous study [46]. It was suggested that higher zeatin levels positively regulate cell division and plant growth under low temperature in barley. However, zeatin concentration in *Az34* mutant has not changed after low temperature treatment. This result indicates that ABA deficiency may negatively affect the signaling of zeatin, whereas the role of cytokines in respond to low temperature remains unclear, and, thus, the related mechanism needs to be further studied.

## 4. Materials and Methods

### 4.1. Experimental Design

We used an ABA-deficient mutant *Az34* (= *nar2a*) and its wild type background, the cultivar Steptoe, both kindly provided by Prof. Ulrich Schaffrath at RWTH Aachen University). Steptoe, CI-15229, is a six-row, rough awned, spring feed barley developed for production in the Pacific Northwest. *Az34* is a molybdenum cofactor mutant of barley that is isogenic to the Steptoe. The mutation affects aldehyde oxidase enzyme activity, which prevents the conversion of abscisic acid aldehyde to abscisic acid, hence decreasing the endogenous ABA level [72]. Seeds were sown in plastic pots filled with 1.2 kg clay soil in growth chambers. Four seeds were sown in each pot and seedlings were cultivated in a climate-controlled greenhouse with a 25/18 °C temperature regime (14/10 h day/night). The light intensity was 450 μmol m^−2^ s^−1^ by sunlight plus metal-halide lamps and the relative humidity was 70 ± 5%. For low temperature stress, one half of the mutant and wild-type seedlings at 3-leaf stage were moved into a low temperature incubator (14/10 h day/night photoperiod) (0 °C, 24 h), while the control plants were kept at optimum temperature. Each treatment included three replicates and each replicate was comprised of 6 pots. The latest fully expanded leaf was immediately harvested for protein extraction, TEM analysis, enzyme activity, and hormone content measurements after a 24-h low temperature treatment. The samples were immediately frozen in liquid nitrogen and stored at −80 °C for further analysis.

### 4.2. Chlorophyll a Fluorescence and Gas Exchange

Chlorophyll *a* fluorescence was measured under control conditions, immediately after the low temperature treatment using an imaging chlorophyll fluorometer (FluorCam 800 MF, PSI, Prague, Czech Republic). Before measuring, plants were dark-adapted for 30 min under low temperature, and the latest fully expanded leaf was imaged. Chlorophyll *a* fluorescence induction curves (OJIP curve) were measured using a Plant Efficiency Analyzer (Pocket-PEA, Hansatech, Norfolk, UK). The collected data were processed by PEA Plus 1.04 (Hansatech, Norfolk, UK). The information about parameters derived from the OJIP transient of chlorophyll fluorescence was shown in Table 5. Stomatal conductance (*g_s_*) and net photosynthetic rate (*A_n_*) were measured on the same leaf used for chlorophyll *a* fluorescence measurements, using a portable photosynthesis system (LI-6400, LI-Cor, Lincoln, NE, USA) with a photo-synthetically active radiation of 1200 μmol m^−2^ s^−1^, and the CO_2_ concentration was set to 400 μmol mol^−1^. Each measurement included six replicates.

### 4.3. Protein Extraction, Quantification and Identification by 4D-Proteomics

Total protein was extracted from the control and cold-treated leaf (the latest fully expanded leaf) of WT and *Az34* mutant plants, and was analyzed by mass spectrometry (Figure S1A). Briefly, the samples (0.25 g) were ground while cooled by liquid nitrogen. A total of 1 mL lysis buffer (8 M urea, 1% Triton-100, 10 mM dithiothreitol, and 1% protease inhibitor (Calbiochem, Darmstadt, Germany)) was added to the centrifuge tube, followed by sonication three times on ice using a high intensity ultrasonic processor (Scientz, Ningbo, China). The remaining debris was removed by centrifugation at 20,000× *g* at 4 °C for 10 min. The protein was precipitated with 20% TCA for 2 h at −20 °C. The pellet was washed three times with cold acetone, and then the pellet was dissolved in 8 M urea and the protein concentration was determined with BCA kit according to the manufacturer’s instructions. After that, the sample was digested with trypsin. The protein solution was reduced with 5 mM dithiothreitol for 30 min at 56 °C and alkylated with 11 mM iodoacetamide for 15 min at room temperature in darkness. Then, the protein sample was diluted by adding 100 mM TEAB to urea concentration less than 2M. Trypsin was added at 1:50 trypsin-to-protein mass ratio for the first digestion overnight and 1:100 trypsin-to-protein mass ratio for a second 4 h-digestion. The tryptic peptides were loaded onto a reversed-phase analytical column (15 cm length and 75 µm inner diameter) coupled to the NanoElute ultra-high performance liquid system. The peptides were subjected to nanospay ionisation followed by tandem mass spectrometry (MS/MS) in a Q ExactiveTM Plus (Thermo Fisher, Waltham, MA, USA) coupled online to a NanoElute UPLC (Bruker Daltonics GmbH, Bremen, Germany). Three independent replicates were performed for each treatment.

### 4.4. Database Search and Bioinformatics

The resulting MS/MS data were processed using Maxquant search engine (v.1.6.6.0). Uniprot *Hordeum vulgare* (Version 2019-12, 189,799 entries) [73] (International Barley Genome Sequencing et al., 2012) was added to the database to calculate the false positive rate (FDR) caused by random matching, and a common contamination library was added to the database to eliminate the influence of contaminated proteins in the identification results. Trypsin was specified as a cleavage enzyme, allowing up to 2 missing cleavages. The minimum length of the peptide segment was set as 7 amino acid residues. The missing match tolerance of the primary parent ion of First match and Main match is set as 40 ppm and 40 ppm, respectively, and the missing match tolerance of the secondary fragment ion is 0.04 Da. The alkylation of cysteine was defined as fixed modification, methionine oxidation, and N-terminal acetylation of protein were defined as variable modifications. Peptide-to-spectrum (PSM) matches and protein identification with false discovery rate (FDR) smaller than 1%. The proteins were identified with the software MoMo (V5.0.2 http://meme-suite.org/tools/momo (accessed on 30 August 2018)) and annotated using GO annotation (v.5.14-53.0 http://www.ebi.ac.uk/interpro/ (accessed on 8 January 2019)); Domain annotation (v.5.14-53.0 http://www.ebi.ac.uk/interpro/ (accessed on 4 January 2017)); Kyoto Encyclopedia of Genes and Genomes (KEGG) annotation with software KAAS (v.2.0 http://www.genome.jp/kaas-bin/kaas-main (accessed on 26 February 2019)) and KEGG Mapper (V2.5 http://www.kegg.jp/kegg/mapper.html); subcellular localization with Wolfpsort (v.0.2 http://www.genscript.com/psort/wolf_psort.html (accessed on 13 May 2010)) and CELLO (v.2.5 http://cello.life.nctu.edu.tw/ (accessed on 9 June 2014)). Enrichment analysis was performed with a Perl module (v.1.31 https://metacpan.org/pod/Text::NSP::Measures::2D::Fisher (accessed on 26 March 2008)) and expression profiles were clustered with the R Package pheatmap (v.2.0.3 https://cran.r-project.org/web/packages/cluster/ (accessed on 9 July 2019)).

### 4.5. Transmission Electron Microscopy for Chloroplast Ultrastructure Analysis

The latest fully expand barley leaf tissues were cut from different treatments with a scalpel and fixed in 2.5% (*v*/*v*) glutaraldehyde in 0.1 M phosphate-buffered saline (pH 7.0) and infiltration under vacuum. The fixed samples were rinsed in 0.1 M sodium cacodylate buffer for 15 min and post-fixed in 1% (*w*/*v*) osmium tetroxide in the same buffer for 2 h at room temperature. Next, the samples were dehydrated in acetone dilutions and embedded in Spi-pon 812 resin. Thin sections (70 nm) were cut with a ultramicrotome (EM UC7, Leica, Weztlar, Germany) and double stained with uranyl acetate and lead citrate before being observed and imaged under a transmission electron microscope (TEM, HT7700, Hitachi, Japan) at 80 kV.

### 4.6. Determination of Hormone Concentration in Barley Leaf

Concentrations of endogenous hormone was performed on an HPLC-MS/MS system consisting of an AB Qtrap6500 triple quadrupole mass spectrometer and an Agilent 1290 HPLC system. The leaf sample (1 g) was ground under liquid nitrogen and transferred to a glass tube. Ten mL extraction buffer (isopropanol/HCl) was added to the powder, followed by shaking for 30 min at 4 °C and then 20 mL dichloromethane (CH_2_Cl_2_) was added, followed by shaking for another 30 min at 4 °C. After centrifugation at 15,000× *g* at 4 °C, the organic phase was transferred to the glass vials and dried with nitrogen under dark conditions. The residue was dissolved with 400 µL of methanol and was filtered through 0.22 µm filters. The solution was analyzed for hormone concentration with the method of ESI-HPLC-MS/MS [70]. The liquid chromatography conditions were as follows: the reverse-phase chromatograph column was poroshell 120 SB-C18 (2.1 × 150, 2.7 µm) and column temperature 30 °C. The injection volume was 2 µL. Mass spectrometry conditions were as follows: the air curtain gas 15 psi, spray voltage is 4500 V, atomization pressure 65 psi, auxiliary gas pressure 70 psi and atomization temperature 400 °C. The specification for quantification of hormones by HPLC-ESI-QQQ-Extractive Focus-MS/MS were listed in Table 6, and the calibration curves of hormone quantification were listed in Table S4. The measurement was performed with three replicates.

### 4.7. Determination of Activity of Enzymes Involved in Carbohydrate and ROS Metabolisms

The activities of key carbohydrate metabolism enzymes were measured following the method of Jammer et al. [30] summarized in Figure S1B. Briefly, the leaf samples (0.5 g) were ground in liquid nitrogen, and then homogenized using 1 mL of extraction buffer (40 mM Tris-HCl pH 7.6, 3 mM MgCl_2_, 1 mM EDTA, 0.1 mM PMSF, 1 mM benzamidine, 14 mM β-mercaptoethanol, 24 μM NADP) and incubated 30 min on ice. The homogenate was centrifuged at 4 °C and 13,200× *g* for 30 min. A fraction of the supernatant was dialyzed overnight with 20 mM potassium phosphate buffer (pH 7.4) at 4 °C for measuring the activities of sucrose synthase (Susy), hexokinase (HXK), fructokinase (FK), vacuolar invertase (vacInv), and cytoplasmic invertase (cytInv). Non-dialyzed supernatant was used for measurements of activities of (fructose 1,6-bisphosphate) aldolase (Ald), phosphofructokinase (PFK), glucose-6-phosphate dehydrogenase (G6PDH), phosphoglucoisomerase (PGI), phosphoglucomutase (PGM), and ADP-glucose pyrophosphorylase (AGPase), and UDP-glucose pyrophosphorylase (UGPase). The pellet was washed three times with pre-cooled distilled water, re-suspended in 1 mL of high salt buffer (1 M NaCl, 40 mM Tris-HCl pH 7.6, 3 mM MgCl_2_, 1 mM EDTA, 0.1 mM PMSF, 1 mM benzamidine, 14 mM β-mercaptoethanol, 24 μM NADP), and then mixed at 4 °C overnight. The homogenate was centrifuged at 4 °C and 13,200× *g* for 30 min, and, subsequently, the supernatant (cell wall extract) was dialyzed overnight with 20 mM potassium phosphate buffer (pH 7.4) at 4 °C for measuring cell wall invertase (cwInv) activity. Susy, HXK, FK, Ald, PFK, G6PDH, PGI, PGM, AGPase, and UGPase were determined in kinetic enzyme assays. The activities of Susy, HXK, FK, PGI, and PGM were calculated by recording the increase in absorbance at 340 nm due to conversion of nicotinamide adenine denucleotide (NAD) to nicotinamide adenine denucleotide (NADH). The AGPase, UGPase, and G6PDH activities were calculated by recording the increase in absorbance at 340 nm due to conversion of nicotinamide adenine denucleotide phosphate (NADP) to NADH. The Ald activity was calculated based on the increase in absorbance at 340 nm due to conversion of NADH to NAD. The PFK activity was calculated depending on the decrease in absorbance at 340 nm due to conversion of NADH to NAD. The adenosine-5′-diphosphoglucose (ADPGlc), fructose-1,6-bisphosphate (F1,6bisP), fructose, glucose-6-phosphate (G6P), glucose, fructose-6-phosphate (F6P), glucose-1-phosphate (G1P), sucrose, and uridine-5′-diposphoglucose (UDPGlc) was used as specific substrates for AGPase, Ald, FK, G6PDH, HXK, PFK, PGI, PGM, Susy, and UGPase measurements, respectively. For the control reactions, the specific substrates were omitted. The invertase (cytInv, vacInv and cwInv) activities were analyzed in the end point assays, and the amount of liberated glucose was determined by a measurement of the absorbance at 405 nm. The sucrose is the specific substrate for the three invertase. For their control reactions, the sucrose was omitted. The measurement was performed on Epoch Microplate Spectrophotometer (Biotek, Bad Friedrichshall, Germany) and each sample was measured with three replicates.

The activities of antioxidant enzymes were measured using the method described by Fimognari et al. [29], summarized in Figure S1C. Briefly, the leaf samples (0.5 g) were ground in liquid frozen and then homogenized using 1 mL of extraction buffer (40 mM Tris HCl pH7.6, 1 mM EDTA, 0.1 mM phenylmethane sulfonyl fluoride, 1 mM Benzamidine, 24 µM NADP, 14 mM β-Mercaptoethanol) and mixed, and the homogenate was incubated 30 min at 4 °C. The homogenate was centrifuged for 10 min at 4 °C with 13,200× *g*. The supernatant was dialyzed overnight with 20 mM phosphate buffer (pH 7.6) for determining the activities of ascorbate peroxidase (APX), catalase (CAT), dehydroascorbate reductase (DHAR), glutathione reductase (GR), glutathione S-transferase (GST), monodehydroascorbate reductase (MDHAR), peroxidase (POX), and superoxide dismutase (SOD). The pellet was washed three times with pre-cooled distilled water, and 1 mL of high salt buffer (40 mM Tris HCl pH7.6, 15 mM EDTA, 3 mM MgCl_2_, and 1 M NaCl) was added to the tubes and mixed in the samples, and subsequently put in the shaker at 4 °C overnight. The homogenate was centrifuged for 10 min at 4 °C with 13,200× *g*, and then the supernatant was dialyzed overnight with 20 mM phosphate buffer (pH 7.4) for determining apoplastic peroxidase (cwPOX) enzyme activity. APX activity was calculated by the oxidation of ascorbate at 290 nm at 30 °C for 40 min. CAT activity was calculated by recording the disappearance of H_2_O_2_ at 240 nm at 30 °C for 40 min. DHAR activity was calculated by recording the formation of ascorbate at 265 nm at 25 °C for 40 min. The GR and MDHAR activities were calculated by recording the disappearance of NADPH at 340 nm at 30 °C for 40 min. The GST activity was calculated by recoding the formation of (2,4-dini-trophenyl) glutathione (DNP-GS) at 334 nm at 30 °C for 40 min. The POX abd cwPOX activities were r calculated by recording the formation of tetraguaiacol at 450 nm at 30 °C for 40 min. The measurements were performed on an Epoch Microplate Spectrophotometer (Biotek, Bad Friedrichshall, Germany), and each sample was measured with three replicates.

### 4.8. Quantifications of Proline and H_2_O_2_

The concentration of proline was determined according to Bates, Waldren, and Teare [74]. Briefly, the fresh leaves were ground with liquid nitrogen, and the powder (0.2 g) was homogenized by 3% sulfosalicylic acid. The supernatant of 2 mL was collected in 15 mL centrifuge tube and added in 2 mL glacial acetic acid and 2 mL ninhydrin acid followed by heating for 30 min. Toluene was added and vortexed for 1 min. The concentration of proline was determined by measuring the absorbance at 520 nm. For H_2_O_2_ determination, the fresh leaf samples were grounded, and 0.1 g powder were homogenized with 0.9 mL acetone, then centrifuged at 8000× *g* for 10 min at 4 °C. The supernatant of 0.25 mL was collected in 1.5 mL centrifuge tube, and the H_2_O_2_ concentration was measured according to the instructions of H_2_O_2_ analysis kit (Youxuan Biotech Ltd., Shanghai, China). The absorbance was recorded at 415 nm and the measurement was performed with three replicates.

### 4.9. Quantifications of Soluble Sugars and Soluble Proteins

The concentration of soluble sugar was measured using the anthrone method [75]. Dry leaf samples were ground, and 0.1 g powder was extracted twice with 80% (*v*/*v*) ethanol at 80 °C for 30 min, using centrifugation at 3000× *g* for 10 min. The supernatant was collected for measuring soluble sugar. The absorbance was read at 620 nm.

Soluble protein was measured by the Coomassie Brilliant Blue G-250 method [76]. Briefly, the fresh leaves were grounded with liquid nitrogen and the powder (0.2 g) was homogenized with 1.8 mL 0.1 M phosphate buffer solution (PBS, PH 7.2), and then centrifuged at 4000× *g* for 10 min at 4 °C. The supernatant of 0.1 mL was collected in 10 mL centrifuge tube with 5 mL Coomassie Brilliant Blue G-250, placing 2 min for measurement of soluble protein concentration. The absorbance was recorded at 595 nm. The measurement was performed with three replicates.

### 4.10. Statistical Analysis

A two-tailed *t* test was employed to test the differentially expressed proteins (*p* < 0.05 and fold change > 1.5 or fold change < 1/1.5) against all identified proteins. Gene Ontology (GO) with a *p* < 0.05 based on Fisher’s exact test is considered significant; and KEGG pathway with a *p* < 0.05 based on Fisher’s exact test is considered significant. The data of enzyme activity, hormone concentration, and gene transcript were subjected to the two-way ANOVA using SPSS 20 (SPSS, Chicago, IL, USA). The difference of activities for a given carbohydrate metabolism enzyme and antioxidant enzyme between treatments was calculated by Microsoft Excel 2010. The heatmaps were visualized by TBtools [27]. Principal component analysis (PCA) based on eigenvalue decomposition of data correlation matrix was performed to present the relationships between ABA and physiological traits and enzyme activities, respectively.

## 5. Conclusions

**Figure 8:**
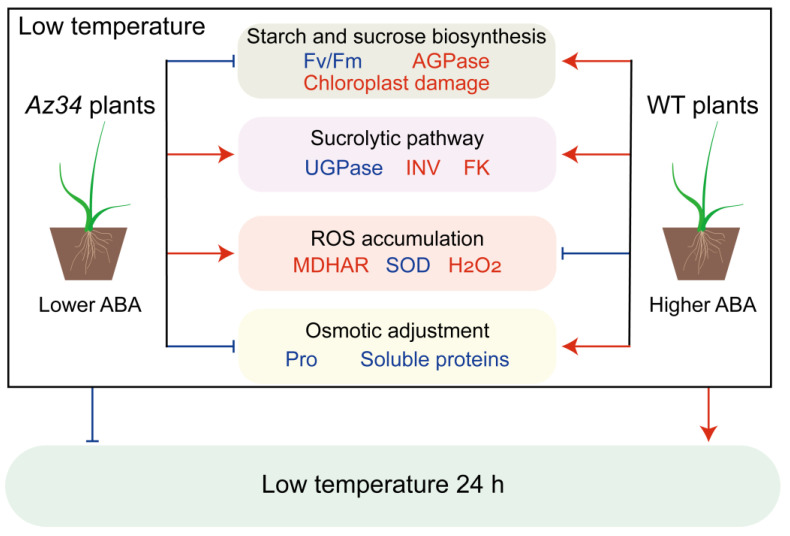
A schematic model of the low ABA and low temperature on barley plants. The blue font indicates that the index is lower in *Az34* plants than in WT plants under low temperature. The red font indicates that the index is higher in *Az34* plants than in WT plants under low temperature. The red arrows and the blue bars indicate that the pathway was facilitated and inhibited under low temperature, respectively. ABA, abscisic acid; Fv/Fm, maximum quantum efficiency of photosystem II; AGPase, ADP-glucose pyrophosphorylase; FK, fructokinase; INV, invertase; MDHAR, monodehydroascorbate reductase; SOD, superoxide dismutase; UGPase, UDP-glucose pyrophosphorylase; WT, wild type.

In conclusion, the results demonstrated that ABA deficiency reduced photosynthesis by damaging chloroplast ultrastructure, and that it caused modifications in starch biosynthesis and sucrose metabolism, thus decreasing the responses to low temperature (Figure 8). For providing further evidence, the DAPs identified by 4D-proteomics between WT and *Az34* indicated that ABA deficiency decreased the abundances of photosynthesis-antenna proteins, photosynthesis related proteins, and glutathione metabolism related proteins. In addition, ABA deficiency increased the ROS accumulation and negatively affected the hormonal regulatory network, demonstrating that the ABA is involved in various metabolic pathways to regulate the low temperature response. The PCA outputs showed that ABA regulated the activities of SOD, AGPase, FK, and MDHAR, hence promoting the low temperature responses. This suggests that ABA deficiency reduced low temperature responses via disturbing stability of chloroplast ultrastructure, modifying the starch and sucrose metabolisms, and affecting antioxidant enzyme activities in barley. These results provided useful information for crop cold cultivation.

## Figures and Tables

**Table 1:** Net photosynthetic rate, stomatal conductance, and fluorescence transient chlorophyll *a* parameters deduced from analysis of the JIP-test for the latest fully expanded leaves of wild type and *Az34* barley under optimum and low temperature.

	Treatment	*An* (µmol CO_2_ m^−2^ s^−1^)	*g_s_* (mmol m^−2^ s^−1^)	φPo	ψEo	φEo	φRo
OT	WT	27.80 ± 0.66 ^a^	271.90 ± 7.86 ^a^	0.77 ± 0.01 ^a^	0.57 ± 0.02 ^ab^	0.44 ± 0.02 ^ab^	0.19 ± 0.01 ^bc^
	*Az34*	27.70 ± 0.60 ^a^	264.67 ± 4.66 ^a^	0.78 ± 0.03 ^a^	0.65 ± 0.08 ^a^	0.51 ± 0.09 ^a^	0.26 ± 0.06 ^ab^
LT	WT	17.50 ± 0.35 ^c^	205.83 ± 8.90 ^b^	0.53 ± 0.07 ^b^	0.60 ± 0.09 ^b^	0.32 ± 0.09 ^b^	0.29 ± 0.04 ^a^
	*Az34*	14.63 ± 0.77 ^b^	234.90 ± 4.74 ^c^	0.62 ± 0.03 ^c^	0.51 ± 0.05 ^ab^	0.32 ± 0.05 ^b^	0.14 ± 0.04 ^c^

OT, optimum temperature; LT, low temperature; *A_n_*, net photosynthetic rate; *g_s_*, stomatal conductance, φPo, maximum quantum yield for primary photochemistry; ψE_O_, probability that an electron moves further than QA; φE_O_, quantum yield for electron transport; φR_O_, quantum yield for reduction of end electron acceptors at the PSI acceptor side. Different small letters in each column indicate significant differences at *p* < 0.05 level. Data are expressed as means ± SEM (n = 4).

**Table 2:** Output of the two-way ANOVA for the key carbohydrate metabolism enzymes in the latest fully expanded leaves as affected by ABA-deficiency and low temperature treatment in barley.

Factor	G6PDH	AGPase	UGPase	HXK	Ald	PGI	PGM	PFK	vacInv	cwInv	cytInv	Susy	FK
F_G_	ns	*	*	ns	**	*	**	ns	***	**	ns	ns	**
LT	**	ns	*	*	**	ns	**	***	***	ns	***	ns	ns
F_G_ × LT	ns	*	*	*	ns	ns	***	ns	***	**	***	ns	ns

ns, non-significant; *, *p* < 0.05; **, *p* < 0.01; ***, *p*< 0.001; F_G_, ABA-deficiency; LT, low temperature. G6PDH, glucose-6-phosphate dehydrogenase; AGPase, ADP-glucose pyrophosphorylase; UGPase, UDP-glucose pyrophosphorylase; HXK, hexokinase; Ald, aldolase; PGI, phosphoglucomutase; PGM, phosphoglucomutase; PFK, phosphofructokinase; vacInv, vacuolar invertase; cwInv, cell wall invertase; cytInv, cytoplasmic invertase; Susy, sucrose synthase; FK, fructokinase.

**Table 3:** Output of the two-way ANOVA for the key anti-oxidant enzymes in the latest fully expanded leaves as affected by ABA-deficiency and low temperature treatment in barley.

Factor	APX	CAT	cwPOX	GR	GST	POX	DHAR	MDHAR	SOD
F_G_	**	ns	ns	ns	ns	ns	ns	***	***
LT	**	ns	ns	ns	ns	ns	*	ns	***
F_G_ × LT	ns	ns	ns	ns	ns	ns	ns	ns	***

ns, non-significant; *, *p* < 0.05; **, *p* < 0.01; ***, *p* < 0.001; F_G_, ABA-deficiency; LT, low temperature. APX, ascorbate peroxidase; CAT, catalase; cwPOX, apoplastic peroxidase; GR, glutathione reductase; GST, glutathione S-transferase; POX, cytoplasmic peroxidase; DHAR, dehydroascorbate reductase; MDHAR, monodehydroascorbate reducate; SOD, superoxide dismutase.

**Table 4:** Output of the two-way ANOVA for the hormones in the latest fully expanded leaves as affected by ABA-deficiency and low temperature treatment in barley.

Factor	ABA	SA	JA	MeSA	IAA	IBA	IPA	Zeatin
F_G_	***	***	***	**	ns	***	ns	***
LT	***	**	***	ns	***	**	***	***
F_G_ × LT	**	***	**	ns	ns	***	***	***

ns, non-significant; **, *p* < 0.01; ***, *p*< 0.001; F_G_, ABA-deficiency; LT, low temperature. ABA, abscisic acid; SA, salicylic acid; JA, jasmonate; MeSA, methyl salicylic acid; IAA, indole-3-acetic acid; IBA, indole-3-butytric acid; IPA, indolepro pionic acid.

**Table 5:** Explanation and formulae in the technical data of OJIP cures (rapid fluorescence transient) and the selected JIP-test parameters used in the present study.

Fluorescence Parameters	Illustrations
F_o_ ≌ F_20 μs_	Minimal fluorescence, when all PSII RCs are open
Fv =F_t_ − F_o_	Variable fluorescence at time t
F_m_ = F_P_	Maximal recorded fluorescence intensity, at the peak P of OJIP
φPo = TRo/ABS = 1 − Fo/F_M_ = Fv/Fm	Maximum quantum yield for PS II primary photochemistry att = 0
ψEo = ET_O_/TR_O_	Probability that an electron moves further than Q_A_
φEo = ET_O_/ABS = (1 − F_O_/F_M_) × (1 − V_J_)	Quantum yield for PS II electron transport at t = 0 (ET)
φRo = RE_O_/ABS	Quantum yield for reduction of end electronacceptors at the PSI acceptor side (RE)

Subscript “o” indicates that the parameters refer to illumination onset, when all reaction centers are assumed to be open.

**Table 6:** Identification of the phytohormones of barley leaves by HPLC-ESI-QQQ-Exactive Focus-MS/MS in negative and positive modes.

Compounds	Ion Model	Parental Ion (M/Z)	Daughter Ion (M/Z)	Fragmentor Voltage (V)	Collision Energy (V)
ABA	Negative	263.1	153.0/204.2	−60	−14/−27
IAA	Positive	176.2	129.8/102.9	65	12/42
IBA	Negative	202	116.0/158.0	−80	−20/−18
IPA	Positive	336.2	204.2/136.3	59	28/32
JA	Negative	209.2	58.9	−54	−16
MeSA	Negative	153.0	121.0/93.1	−131	−24/−39
SA	Negative	137	92.6/65	−50	−20/−39
Zeatin	Positive	220.4	136.0/147.9	92	22/16

## Data Availability

All data supporting the findings of this study are available within the paper and within its Supplementary Materials published online. The mass spectrometry proteomics data are available via ProteomeXchange with identifier PXD043642.

## Supplementary Materials

The supporting information can be downloaded at: https://www.mdpi.com/article/10.3390/ijms241411348/s1.

## Abbreviations

ABA: abscisic acid; Ald, aldolase; AGPase, ADP-glucose pyrophosphorylase; APX, ascorbate peroxidase; CAT, catalase; cwInv, cell wall invertase; cwPOX, apoplastic peroxidase; cytInv, cytoplasmic invertase; DAP: differentially abundant proteins; DHAR, dehydroascorbate reductase; FK, fructokinase; G6PDH, glucose-6-phosphate dehydrogenase; GR, glutathione reductase; GST, glutathione S-transferase; HXK, hexokinase; IAA, indole-3-acetic acid; IBA, indole-3-butytric acid; IPA, indole pionic acid; JA, jasmonate; MDHAR, monodehydroascorbate reducate; MeSA, methyl salicylic acid; PFK, phosphofructokinase; PGI, phosphoglucomutase; PGM, phosphoglucomutase; POX, cytoplasmic peroxidase; SA, salicylic acid; SOD, superoxide dismutase; Susy, sucrose synthase; vacInv, vacuolar invertase.
