# Supplementary material for: Abscisic Acid Regulates Carbohydrate Metabolism, Redox Homeostasis and Hormonal Regulation to Enhance Cold Tolerance in Spring Barley
Source: Int J Mol Sci. 2023 Jul 12;24(14):11348. doi: 10.3390/ijms241411348 (PMC10379442; doi:10.3390/ijms241411348)
Supplement: Supplementary file 1 [file ijms-24-11348-s001.zip › SUPPL FIG.pdf]

Figure S1 Diagrammatic illustration for analysis of key carbohydrate metabolism enzymes activity (A), antioxidant enzymes activity (B) and 4D-proteomics (C).

Figure S2 SDS-PAGE of total proteins extracted from the last fully expanded leaf of wild type (WT) and *Az34* barley under optimum and low temperature.

Figure S3 Subcellular localization of the differentially expressed proteins for the comparisons of WT-LT vs WT-OT, *Az34*-LT vs WT-LT, *Az34*-OT vs WT-OT, and *Az34*-LT vs *Az34*-OT. (A) Subcellular localization of the differentially expressed proteins in the comparison of WT-LT vs WT-OT; (B) Subcellular localization of the differentially expressed proteins in the comparison of *Az34*-LT vs WT-LT; (C) Subcellular localization of the differentially expressed proteins in the comparison of *Az34*-OT vs WT-OT; (D) Subcellular localization of the differentially expressed proteins in the comparison of *Az34*-LT vs *Az34*-OT. OT, optimum temperature; LT, low temperature.

Figure S4 GO terms of the differentially expressed proteins for the comparisons of WT-LT vs WT-OT, *Az34*-OT vs WT-OT, *Az34*-LT vs WT-LT and *Az34*-LT vs *Az34*-OT. (A) GO terms of the differentially expressed proteins for the comparison of WT-LT vs WT-OT; (B) GO terms of the differentially expressed proteins for the comparison of *Az34*-OT vs WT-OT; (C) GO terms of the differentially expressed proteins for the comparison of *Az34*-LT vs WT-LT; (D) GO terms of the differentially expressed proteins for the comparison of *Az34*-LT vs *Az34*-OT. OT, optimum temperature; LT, low temperature.

Figure S5 Functional enrichment cluster of the differentially expressed proteins for the comparisons of WT-LT vs WT-OT, *Az34*-LT vs WT-LT, *Az34*-OT vs WT-OT, and *Az34*-LT vs *Az34*-OT. OT, optimum temperature; LT, low temperature.

Figure S6 Activities of key carbohydrate metabolism enzymes in the last fully expanded leaf of wild

type (WT) and *Az34* barley under optimum and low temperature. Ald, aldolase; UGPase, UDP-glucose pyrophosphorylase; Susy, sucrose synthase; AGPase, ADP-glucose pyrophosphorylase; HXK, hexokinase; PGI, phosphoglucomutase; G6PDH, glucose-6-phosphate dehydrogenase; PFK, phosphofructokinase; PGM, phosphoglucomutase; FK, fructokinase; vacInv, vacuolar invertase; cytInv, cytoplasmic invertase; cwInv, cell wall invertase. Different small letters indicate significant differences at $P<0.05$ level. Data are expressed as means ± SEM (n=3).

Figure S7 Activities of antioxidant enzymes in the last fully expanded leaf of wild type (WT) and *Az34* barley under optimum and low temperature. SOD, superoxide dismutase; CAT, catalase; APX, ascorbate peroxidase; POX, cytoplasmic peroxidase; cwPOX, apoplastic peroxidase; GST, glutathione S-transferase; MDHAR, monodehydroascorbate reducate; GR, glutathione reductase; DHAR, dehydroascorbate reductase. Different small letters indicate significant differences at $P<0.05$ level. Data are expressed as means ± SEM (n=3).

Figure S8 Concentrations of soluble sugar (A), soluble protein (B), proline (C) and H_2O_2 (D) in the last fully expand leaf of wild type and *Az34* barley under optimum temperature and low temperature. OT: optimum temperature, LT: low temperature. Different small letters indicate significant differences at $P<0.05$ level. Data are expressed as means ± SEM (n=3).

Figure S9 Biplot of principal component analysis (PCA) of ABA with physiological traits and ABA with enzyme activities performed separately in WT and *Az34* plants under optimum temperature and low temperature. (A) Biplot of PCA of ABA with physiological traits in WT and *Az34* plants under optimum temperature and low temperature; (B) Biplot of PCA of ABA with enzyme activities performed separately in WT and *Az34* plants under optimum temperature and low temperature. ABA, abscisic acid; *An*, net photosynthetic rate; g_s, stomatal conductance; φP_O, maximum quantum yield

for primary photochemistry; ψE_O, probability that an electron moves further than Q_A; φE_O, quantum yield for electron transport; φR_O, quantum yield for reduction of end electron acceptors at the PSI acceptor side. F_V/F_M, maximum quantum efficiency of photosystem II; AGPase, ADP-glucose pyrophosphorylase; UGPase, UDP-glucose pyrophosphorylase; FK, fructokinase; vacInv, vacuolar invertase; cytInv, cytoplasmic invertase; cwInv, cell wall invertase; SOD, superoxide dismutase; MDHAR, monodehydroascorbate reductase.

Figure S1

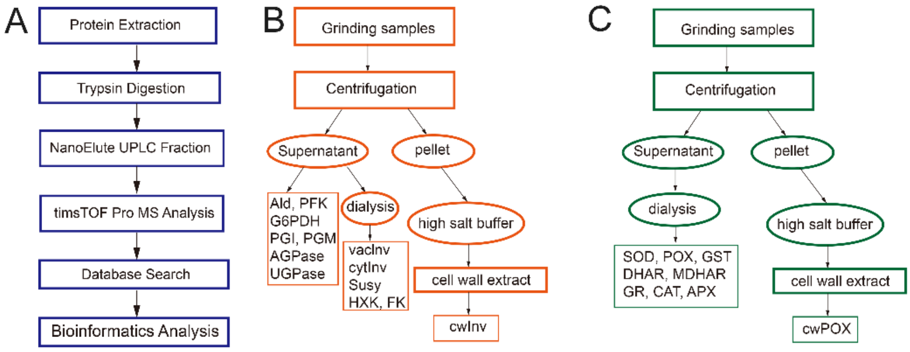
A
Protein Extraction
Trypsin Digestion
NanoElute UPLC Fraction
timsTOF Pro MS Analysis
Database Search
Bioinformatics Analysis
B
Grinding samples
Centrifugation
Supernatant
pellet
Ald, PFK
G6PDH
PGI, PGM
AGPase
UGPase
dialysis
vacInv
cytInv
Susy
HXK, FK
high salt buffer
cell wall extract
cwInv
C
Grinding samples
Centrifugation
Supernatant
pellet
dialysis
SOD, POX, GST
DHAR, MDHAR
GR, CAT, APX
high salt buffer
cell wall extract
cwPOX

Figure S2

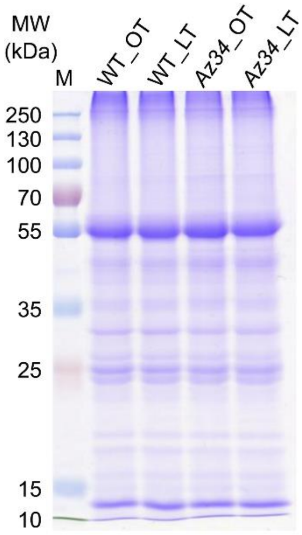

Figure S3

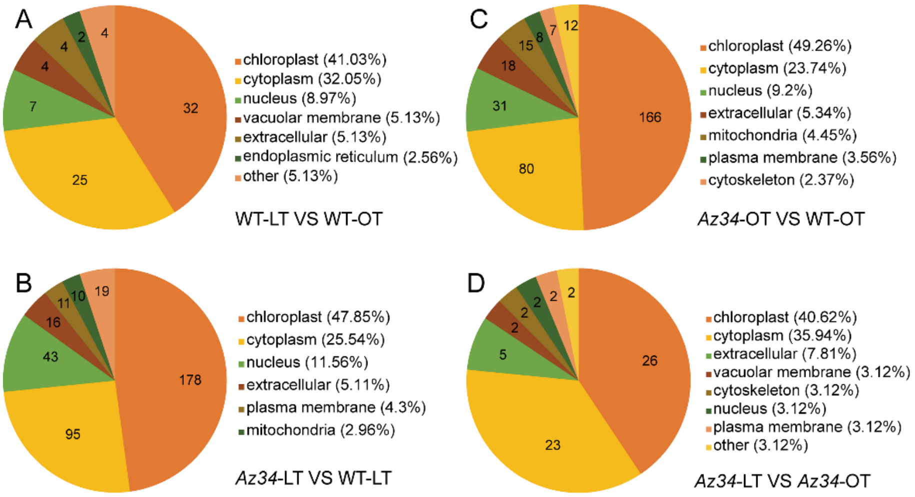
A
32
25
7
4
4
2
4
chloroplast (41.03%)
cytoplasm (32.05%)
nucleus (8.97%)
vacuolar membrane (5.13%)
extracellular (5.13%)
endoplasmic reticulum (2.56%)
other (5.13%)
WT-LT VS WT-OT
C
166
80
31
18
15
8
7
12
chloroplast (49.26%)
cytoplasm (23.74%)
nucleus (9.2%)
extracellular (5.34%)
mitochondria (4.45%)
plasma membrane (3.56%)
cytoskeleton (2.37%)
Az34-OT VS WT-OT
B
178
95
43
16
11
10
19
chloroplast (47.85%)
cytoplasm (25.54%)
nucleus (11.56%)
extracellular (5.11%)
plasma membrane (4.3%)
mitochondria (2.96%)
Az34-LT VS WT-LT
D
26
23
5
2
2
2
2
2
chloroplast (40.62%)
cytoplasm (35.94%)
extracellular (7.81%)
vacuolar membrane (3.12%)
cytoskeleton (3.12%)
nucleus (3.12%)
plasma membrane (3.12%)
other (3.12%)
Az34-LT VS *Az34*-OT

Figure S4

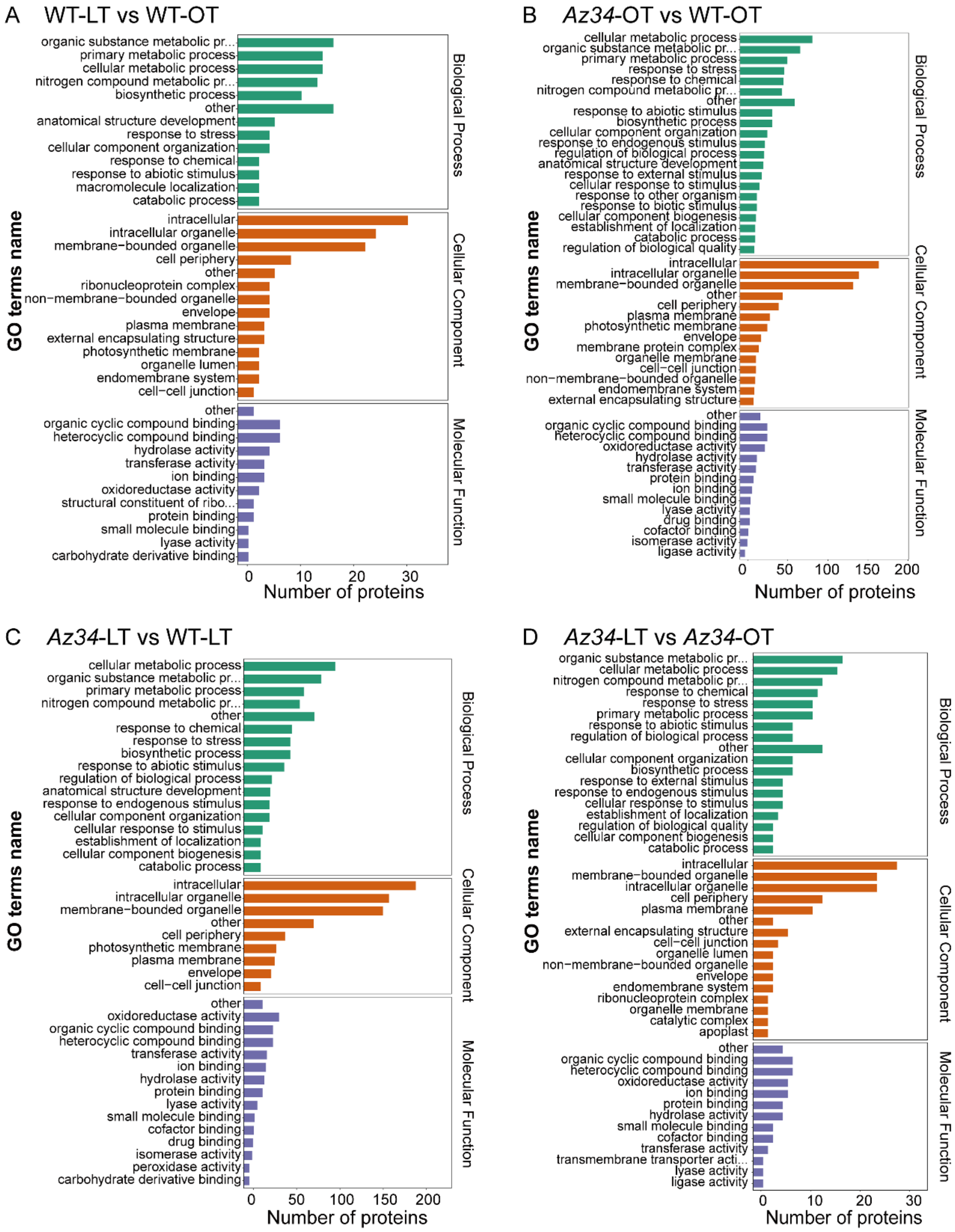

A WT-LT vs WT-OT
B Az34-OT vs WT-OT
C Az34-LT vs WT-LT
D Az34-LT vs Az34-OT
GO terms name
Number of proteins
Biological Process
Cellular Component
Molecular Function

Figure S5

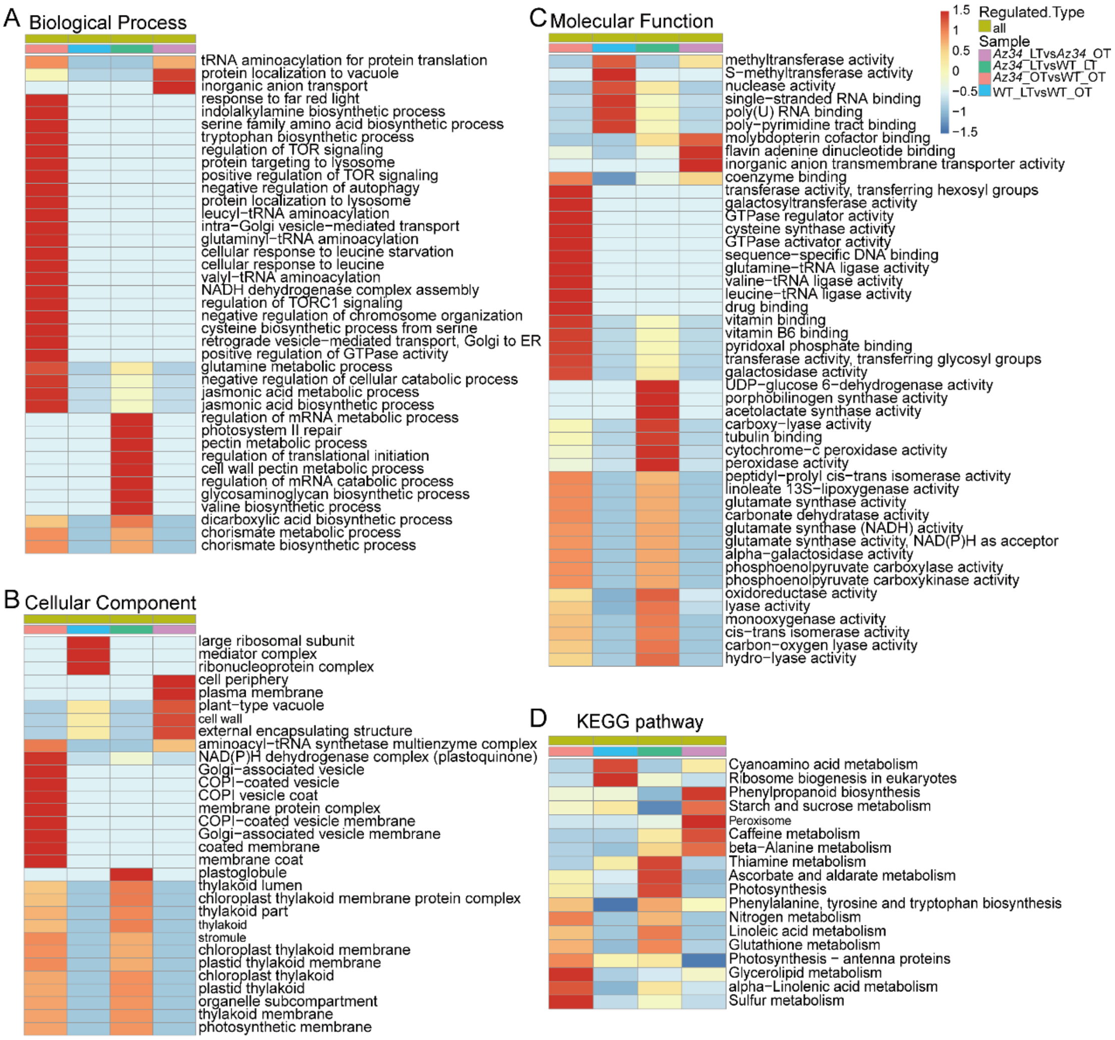

A Biological Process
tRNA aminoacylation for protein translation
protein localization to vacuole
inorganic anion transport
response to far red light
indolalkylamine biosynthetic process
serine family amino acid biosynthetic process
tryptophan biosynthetic process
regulation of TOR signaling
protein targeting to lysosome
positive regulation of TOR signaling
negative regulation of autophagy
protein localization to lysosome
leucyl−tRNA aminoacylation
intra−Golgi vesicle−mediated transport
glutaminyl−tRNA aminoacylation
cellular response to leucine starvation
cellular response to leucine
valyl−tRNA aminoacylation
NADH dehydrogenase complex assembly
regulation of TORC1 signaling
negative regulation of chromosome organization
cysteine biosynthetic process from serine
retrograde vesicle−mediated transport, Golgi to ER
positive regulation of GTPase activity
glutamine metabolic process
negative regulation of cellular catabolic process
jasmonic acid metabolic process
jasmonic acid biosynthetic process
regulation of mRNA metabolic process
photosystem II repair
pectin metabolic process
regulation of translational initiation
cell wall pectin metabolic process
regulation of mRNA catabolic process
glycosaminoglycan biosynthetic process
valine biosynthetic process
dicarboxylic acid biosynthetic process
chorismate metabolic process
chorismate biosynthetic process
B Cellular Component
large ribosomal subunit
mediator complex
ribonucleoprotein complex
cell periphery
plasma membrane
plant−type vacuole
cell wall
external encapsulating structure
aminoacyl−tRNA synthetase multienzyme complex
NAD(P)H dehydrogenase complex (plastoquinone)
Golgi−associated vesicle
COPI−coated vesicle
COPI vesicle coat
membrane protein complex
COPI−coated vesicle membrane
Golgi−associated vesicle membrane
coated membrane
membrane coat
plastoglobule
thylakoid lumen
chloroplast thylakoid membrane protein complex
thylakoid part
thylakoid
stromule
chloroplast thylakoid membrane
plastid thylakoid membrane
chloroplast thylakoid
plastid thylakoid
organelle subcompartment
thylakoid membrane
photosynthetic membrane
C Molecular Function
methyltransferase activity
S−methyltransferase activity
nuclease activity
single−stranded RNA binding
poly(U) RNA binding
poly−pyrimidine tract binding
molybdopterin cofactor binding
flavin adenine dinucleotide binding
inorganic anion transmembrane transporter activity
coenzyme binding
transferase activity, transferring hexosyl groups
galactosyltransferase activity
GTPase regulator activity
cysteine synthase activity
GTPase activator activity
sequence−specific DNA binding
glutamine−tRNA ligase activity
valine−tRNA ligase activity
leucine−tRNA ligase activity
drug binding
vitamin binding
vitamin B6 binding
pyridoxal phosphate binding
transferase activity, transferring glycosyl groups
galactosidase activity
UDP−glucose 6−dehydrogenase activity
porphobilinogen synthase activity
acetolactate synthase activity
carboxy−lyase activity
tubulin binding
cytochrome−c peroxidase activity
peroxidase activity
peptidyl−prolyl cis−trans isomerase activity
linoleate 13S−lipoxygenase activity
glutamate synthase activity
carbonate dehydratase activity
glutamate synthase (NADH) activity
glutamate synthase activity, NAD(P)H as acceptor
alpha−galactosidase activity
phosphoenolpyruvate carboxylase activity
phosphoenolpyruvate carboxykinase activity
oxidoreductase activity
lyase activity
monooxygenase activity
cis−trans isomerase activity
carbon−oxygen lyase activity
hydro−lyase activity
1.5
1
0.5
0
−0.5
−1
−1.5
Regulated.Type
all
Sample
Az34_LTvsAz34_OT
Az34_LTvsWT_LT
Az34_OTvsWT_OT
WT_LTvsWT_OT
D KEGG pathway
Cyanoamino acid metabolism
Ribosome biogenesis in eukaryotes
Phenylpropanoid biosynthesis
Starch and sucrose metabolism
Peroxisome
Caffeine metabolism
beta−Alanine metabolism
Thiamine metabolism
Ascorbate and aldarate metabolism
Photosynthesis
Phenylalanine, tyrosine and tryptophan biosynthesis
Nitrogen metabolism
Linoleic acid metabolism
Glutathione metabolism
Photosynthesis − antenna proteins
Glycerolipid metabolism
alpha−Linolenic acid metabolism
Sulfur metabolism

Figure S6

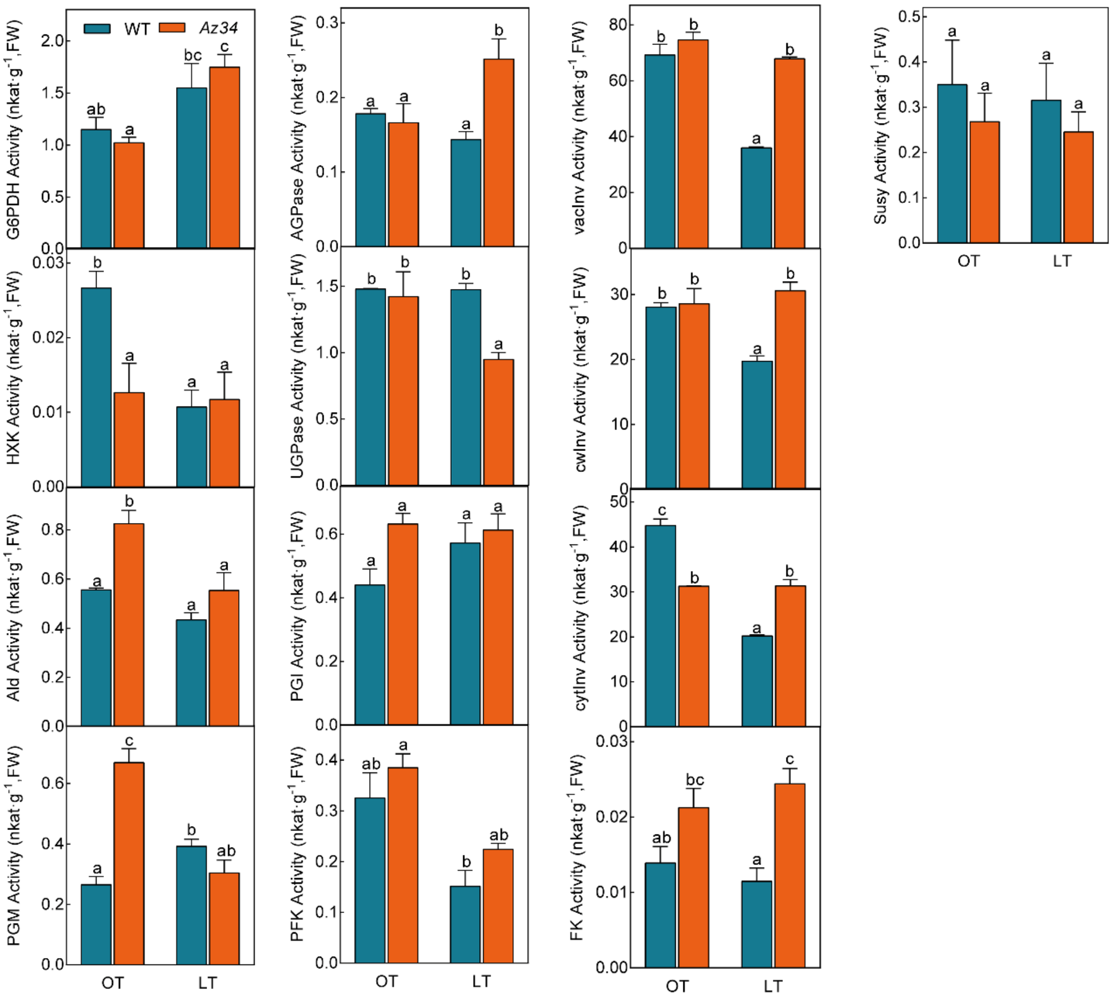
WT
Az34
G6PDH Activity (nkat·g-1,FW)
AGPase Activity (nkat·g-1,FW)
vacInv Activity (nkat·g-1,FW)
Susy Activity (nkat·g-1,FW)
HXK Activity (nkat·g-1,FW)
UGPase Activity (nkat·g-1,FW)
cwInv Activity (nkat·g-1,FW)
Ald Activity (nkat·g-1,FW)
PGI Activity (nkat·g-1,FW)
cytInv Activity (nkat·g-1,FW)
PGM Activity (nkat·g-1,FW)
PFK Activity (nkat·g-1,FW)
FK Activity (nkat·g-1,FW)
OT
LT

Figure S7

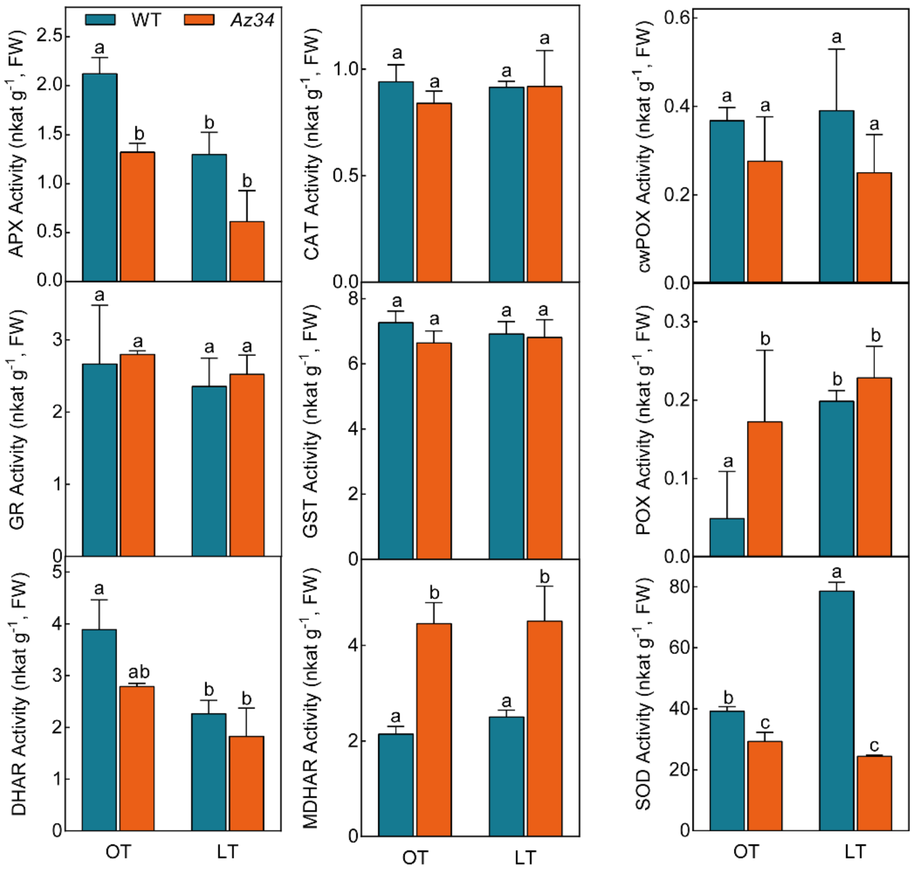

WT
Az34
APX Activity (nkat g^{-1}, FW)
CAT Activity (nkat g^{-1}, FW)
cwPOX Activity (nkat g^{-1}, FW)
GR Activity (nkat g^{-1}, FW)
GST Activity (nkat g^{-1}, FW)
POX Activity (nkat g^{-1}, FW)
DHAR Activity (nkat g^{-1}, FW)
MDHAR Activity (nkat g^{-1}, FW)
SOD Activity (nkat g^{-1}, FW)
OT
LT

Figure S8

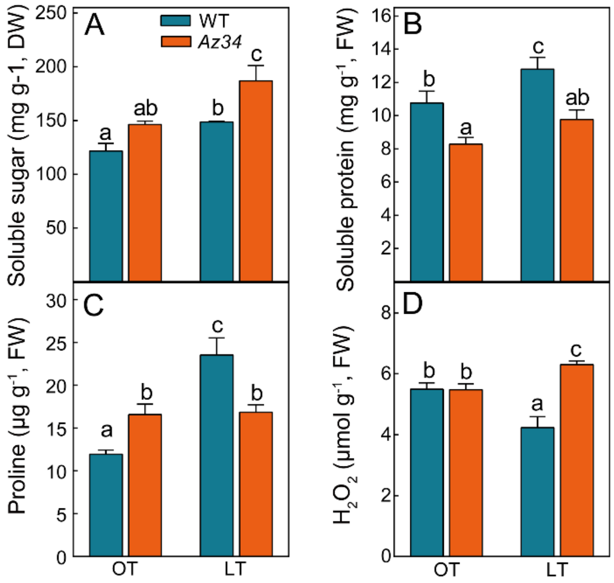

A
B
C
D
WT
Az34
Soluble sugar (mg g-1, DW)
Soluble protein (mg g-1, FW)
Proline (µg g-1, FW)
H2O2 (µmol g-1, FW)
OT
LT
a
ab
b
c

Figure S9

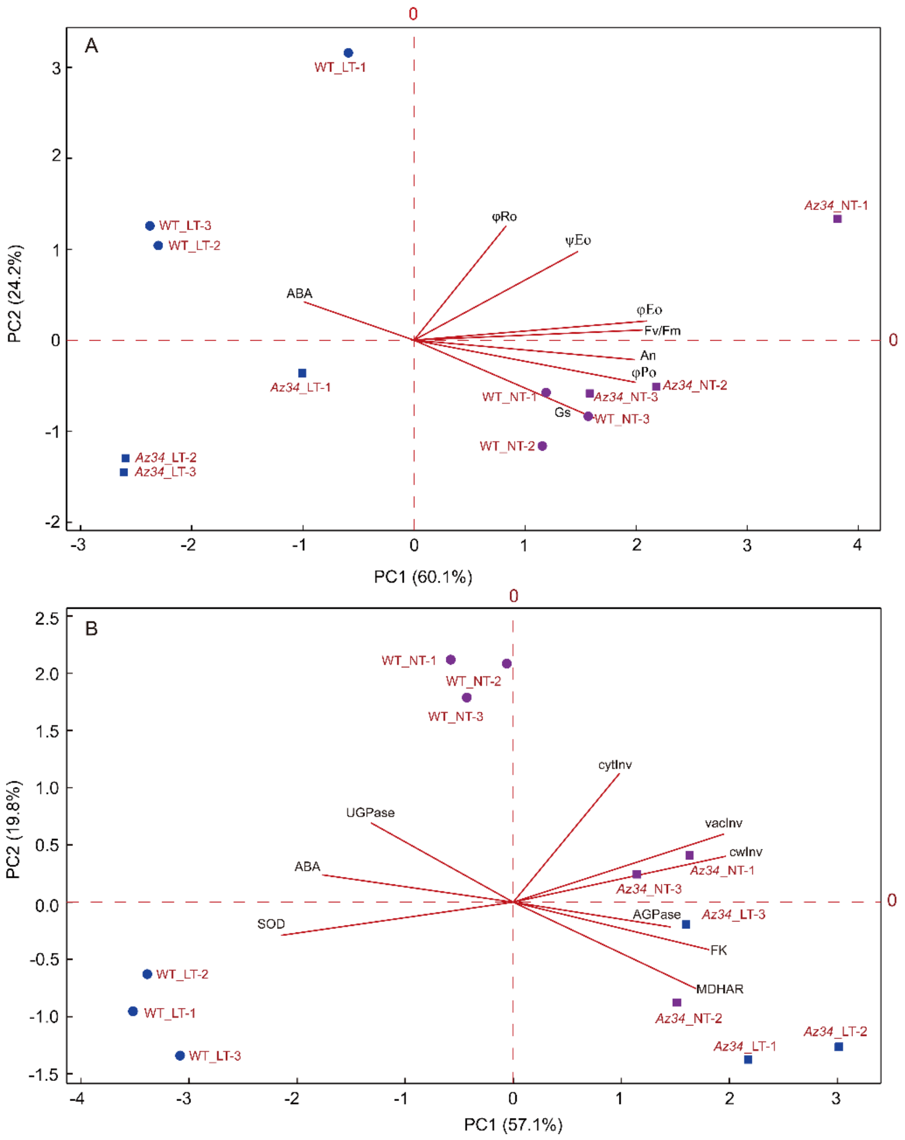
A
WT_LT-1
WT_LT-3
WT_LT-2
ABA
φRo
ψEo
φEo
Fv/Fm
An
φPo
Gs
Az34_NT-1
Az34_NT-2
Az34_NT-3
WT_NT-1
WT_NT-2
WT_NT-3
Az34_LT-1
Az34_LT-2
Az34_LT-3
PC2 (24.2%)
PC1 (60.1%)
B
WT_NT-1
WT_NT-2
WT_NT-3
cytInv
UGPase
vacInv
cwInv
ABA
SOD
AGPase
FK
MDHAR
Az34_NT-1
Az34_NT-2
Az34_NT-3
Az34_LT-1
Az34_LT-2
Az34_LT-3
WT_LT-1
WT_LT-2
WT_LT-3
PC2 (19.8%)
PC1 (57.1%)
