# Supplementary material for: Abscisic Acid Regulates Carbohydrate Metabolism, Redox Homeostasis and Hormonal Regulation to Enhance Cold Tolerance in Spring Barley
Source: Int J Mol Sci. 2023 Jul 12;24(14):11348. doi: 10.3390/ijms241411348 (PMC10379442; doi:10.3390/ijms241411348)
Supplement: Supplementary file 1 [file ijms-24-11348-s001.zip › SUPPL Table.pdf]

Table S1. MS/MS spectrum database search analysis summary

Total spectrum	Matched spectrum	Peptides	Unique peptides	Identified proteins	Quantifiable proteins
2061695	226165 (11.0%)	24722	21770	3879	3200

Table S4. List all of the differently abundant proteins between the four comparable groups (WT_LT/WT_OT, *Az34*_LT/WT_OT, *Az34*_LT/*Az34*_OT, *Az34*_OT/WT_OT). WO1, WT_OT-1; WO2, WT_OT-2; WO3, WT_OT-3; WL1, WT_LT-1; WL2, WT_LT-2; WL3, WT_LT-3; AO1, *Az34*_OT-1; AO2, *Az34*_OT-2; AO3, *Az34*_OT-3; AL1, *Az34*_LT-1; AL2, *Az34*_LT-2; AL3, *Az34*_LT-3; OT, optimum temperature; LT, low temperature.

Table S3. The calibration curve of phytohormone quantification in barley leaves

Compounds	Calibration curve	Correlation coefficient R
ABA	y = 17606.93313 x + 1648.63773	r = 0.99165
IAA	y = 19126.28403 x + 3720.99540	r = 0.9950
IBA	y = 493.11550 x + (- 805.69827)	r = 0.99379
IPA	y = 3.20808e5 x + 19022.90460	r = 0.99183
JA	y = 1208.45440 x + (-1.54468)	r = 0.99817
MeSA	y = 4.57663e5 x + (-1113.60729)	r = 0.99798
SA	y = 415.65675 x + 772.64317	r = 0.99608
Zeatin	y = 1922.94177 x + (-120.11671)	r = 0.99776
